# Impact of mothers' early life exposure to low or high folate on progeny outcome and DNA methylation patterns

**DOI:** 10.1093/eep/dvaa018

**Published:** 2020-11-18

**Authors:** Lundi Ly, Donovan Chan, Mylène Landry, Camille Angle, Josée Martel, Jacquetta Trasler

**Affiliations:** d1 Department of Human Genetics, McGill University, Montreal, QC, Canada; d2 Research Institute of the McGill University Health Centre, Montreal, QC, Canada; d3 Department of Pharmacology & Therapeutics, McGill University, Montreal, QC, Canada; d4 Department of Pediatrics, McGill University, Montreal, QC, Canada

**Keywords:** folic acid, maternal effects, DNA methylation, histone methylation, developmental programming, DOHaD, epigenetics, intergenerational effects, oocyte

## Abstract

The dynamic patterning of DNA and histone methylation during oocyte development presents a potentially susceptible time for epigenetic disruption due to early life environmental exposure of future mothers. We investigated whether maternal exposure to folic acid deficient and supplemented diets starting *in utero* could affect oocytes and cause adverse developmental and epigenetic effects in next generation progeny. Female BALB/c mice (F0) were placed on one of four amino acid defined diets for 4 weeks before pregnancy and throughout gestation and lactation: folic acid control (rodent recommended daily intake; Ctrl), 7-fold folic acid deficient, 10-fold folic acid supplemented or 20-fold folic acid supplemented diets. F1 female pups were weaned onto Ctrl diets, mated to produce the F2 generation and the F2 offspring were examined at E18.5 for developmental and epigenetic abnormalities. Resorption rates were increased and litter sizes decreased amongst F2 E18.5-day litters in the 20-fold folic acid supplemented group. Increases in abnormal embryo outcomes were observed in all three folic acid deficient and supplemented groups. Subtle genome-wide DNA methylation alterations were found in the placentas and brains of F2 offspring in the 7-fold folic acid deficient , 10-fold folic acid supplemented and 20-fold folic acid supplemented groups; in contrast, global and imprinted gene methylation were not affected. The findings show that early life female environmental exposures to both low and high folate prior to oocyte maturation can compromise oocyte quality, adversely affecting offspring of the next generation, in part by altering DNA methylation patterns.

## Introduction

Folic acid supplementation for the prevention of neural tube defects (NTDs) has been well established through randomized trials, with a reported 93% reduction in NTDs [1, 2]. Consequently, in many countries, the food supply is currently fortified with folic acid and periconceptional folic acid supplementation (≥0.4 mg/day) is recommended for women of reproductive age [3, 4]. While high doses of folic acid of 4–5 mg/day are often used for women at elevated risk for NTDs, concerns have been raised regarding the safety of high dose folic acid supplements in terms of offspring health [5]. The great success of folic acid fortification measures across North America has led to women exceeding the recommended daily intakes. For instance, in a recent Canadian study, 90.4% of surveyed pregnant women exceeded the upper limit of 1 mg/day, adding to the concern [6]. However, at the same time, it should be noted that a subpopulation (6.7%) of this Canadian cohort had folate intakes below the Estimated Average Requirements. Similarly, in a recent study from the USA, 21.2% of children and adults studied were reported to have inadequate folate levels with increased risks associated with low economic status [7]. Thus, despite folic acid supplementation programmes, conditions of both low and high folate occur within the North American population.

The vitamin folate or its synthetic form used in supplements, folic acid, is important for one-carbon metabolism and biological methylation reactions including those involved in the key epigenetic modifications of DNA and histone methylation. Thus, exposures of females to low or high folate have the potential to impact epigenetic patterning in their offspring during development. A number of studies have found evidence of epigenetic perturbations in somatic tissues of offspring exposed to low or high folate during gestation. For example, mouse and rat studies have examined the effects of moderate to high dose supplementation (2.0- to 20-fold rodent daily recommended intake) and reported altered DNA methylation patterns in offspring somatic tissues, including the brain [8–12]. In a large human study, increased maternal plasma folate status was linked to decreased DNA methylation levels in cord blood [13]. In contrast, tissues that have received little attention regarding low or high folate exposures during gestation are the germ cells of the embryo and fetus. The epigenome, including both DNA and histone methylation, is reprogrammed in the embryonic gonad and thus is likely to represent one of the most susceptible tissues to the induction of epigenetic defects [14, 15]. Furthermore, altered epigenetic profiles in germ cells have the potential to be transmitted by mature oocytes or sperm and result in intergenerational effects.

A few studies have examined the epigenetic impact of low and or high folate on developing male germ cells. Paternal lifetime folate deficiency, starting *in utero* and continuing postnatally, in a mouse model, resulted in altered histone and DNA methylation in sperm and led to increased birth defects in the next generation offspring [16]. In a previous study, we showed that a similar lifetime exposure to both folic acid deficient (FD) and supplemented (FS) diets in males was associated with altered DNA methylation in sperm and adverse effects in the next generation offspring, including increased death in the early postnatal period and altered epigenetic patterning in somatic tissues [12]. Together, these studies indicate that gestational exposures to low or high folate can affect epigenetic patterning in germ cells with intergenerational consequences.

Like sperm, oocytes transmit key epigenetic information from parent to offspring. As in male germ cells, DNA methylation patterns are, for the most part, erased in female primordial germ cells at mid gestation [15]. However, while in males DNA methylation reacquisition begins in the prenatal gonad, in the female, DNA methylation patterns in germ cells are only acquired after birth when oocytes enter the growth phase preceding ovulation [17, 18]. Histone methylation dynamics during female germ cell development are complex with certain marks, such as histone 3 lysine 36 trimethylation (H3K36me3), preceding and necessary for DNA methylation, and evidence of interactions between different types of histone methylation marks [18–23]. Thus, it is likely that the timing during germ cell development for female and male germ cells to be susceptible to the effects of low and high folate will be different.

A few studies suggest that early female germ cells will be susceptible to the induction of epigenetic alterations by differing gestational environments. For the well-studied agouti viable yellow locus, when females of the F0 generation were given methyl donors on days 8.5–15.5 of gestation, a shift of coat colour, associated with increased methylation of the intracisternal a particle element found at the locus, was seen in F2 offspring of the F1 dams [24, 25] The results indicate that DNA methylation, at least at a single locus, can be influenced by methyl donor supply in early female germ cells. In an additional example, involving impaired folate metabolism (methionine synthase reductase or *Mtrr* mutation) rather than diet, there was evidence of maternal grandparental transmission of epigenetic instability and birth defects across several generations [26].

The rationale for this study was based on accumulating evidence that women are exposed to too little and too much folic acid during pregnancy and that both types of exposures can impact the epigenome and potentially be passed on to the next generation. Here, our goal was to use a well characterized mouse model to examine the intergenerational impact of both low and high folate on early female germ cells.

## Methods

### Mice and Diets

All procedures respected the Canadian Council on Animal Care and the study was approved by the McGill University Animal Care Committee. Animals were housed at the Research Institute of the McGill University Health Centre pathogen-free animal facility under a 12 hour Light: 12 hour Dark cycle in a temperature and humidity-controlled environment with *ad libitum* food and water.

**Figure 1.**
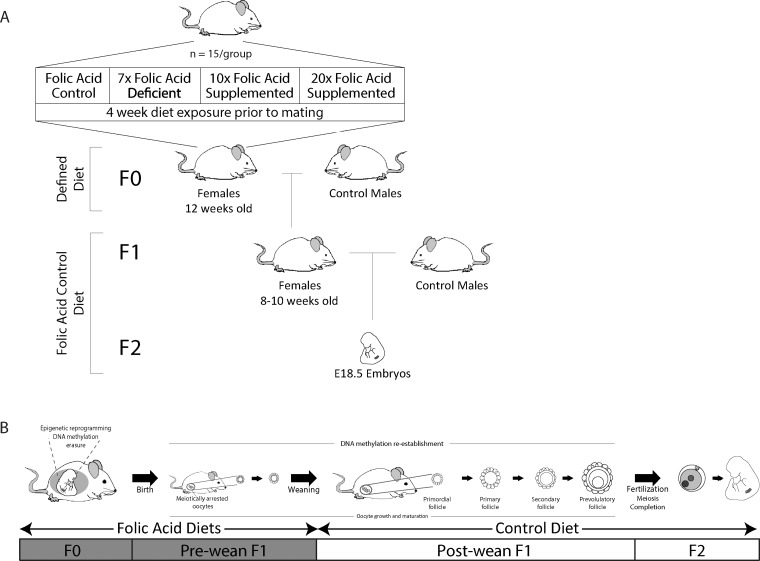
Study design for effects of prenatal female exposure to folate deficiency and folic acid supplementation. (A) Scheme outlining times of exposure to folic acid diets at each generation. BALB/c F0 ♀ (*n* = 15) were exposed to one of four defined diets: folic acid control diet (Ctrl; 2 mg/kg folic acid), 7-fold folic acid deficient diet (7FD; 0.3 mg/kg), 10-fold folic acid supplemented diet (10FS; 20 mg/kg) or 20-fold folic acid supplemented diet (20FS; 40 mg/kg). F1 females were weaned onto their Ctrl diets. (B) Outline of folic acid exposures and coinciding epigenetic events in female germ cell development.

Figure 1 outlines the diet exposures and breeding schemes. Eight-week-old (F0) female BALB/c mice (n = 15/group) (Charles River, Canada) were fed one of four amino-acid defined diets (Harlan Teklad, USA) for 4 weeks prior to mating: folic acid control (Ctrl; 2 mg/kg diet) containing the rodent recommended daily intake of folic acid for mice [27], 7-fold folic acid deficient (7FD; 0.3 mg/kg diet), 10-fold folic acid supplemented (10FS; 20 mg/kg diet) or 20-fold folic acid supplemented (20FS; 40 mg/kg diet) diets. *De novo* synthesis of folate by intestinal bacteria was prevented by supplementing diets with 1% succinylsulfathiazole. These same diets have been used in other studies of developmental effects of folate deficiency and supplementation [12, 16, 28–30]. After 4 weeks on the diets, females were mated with sexually mature, 10-week-old F0 male BALB/c mice maintained on regular mouse chow diets (Product #2018, Harlan Teklad, USA). Throughout mating, gestation and lactation, the F0 females remained on their respective diets until the F1 female offspring were weaned at postnatal day (PND) 20 onto Ctrl diets.

At 8–10 weeks of age, 54 F1 females, representative of 10 Ctrl, 11 FD, 10 10FS and 9 20FS different original F0 litters per diet group, were mated with 10-week-old male BALB/c mice fed regular mouse chow. Throughout mating and gestation, the F1 females were fed the folic acid control diet (Ctrl; 2 mg/kg diet) (Harlan Teklad, USA). The presence of a vaginal plug on the morning after mating was designated as 0.5 days post coitum (dpc). At E18.5, ovaries from the F1 females were collected to determine the number of ovulation sites by counting corpora lutea (CL). Uterine horns were removed and opened to assess implantation by counting viable embryos and resorption sites. Pre-implantation loss was calculated for each female as the difference between the number of CLs and implantation sites. The difference between the numbers of implantation and resorption sites was used as a measure of post-implantation loss. Placentas, viable embryos and resorption sites were removed and weighed. Embryos were sexed by anogenital distance and later verified by sexing PCR to ensure accuracy as described in [31], their crown-rump length measured and evaluation of gross morphological abnormalities performed. Referring to ‘The Atlas of Mouse Development’ [32], embryos and late resorptions were examined for developmental delay and malformations such as cleft palate, closed eyelids, pointy nose, thick neck, curved tail, back and limb malformations. Embryos that deviated by 2 SDs from the means of the mean embryo weights, calculated per litter, across the Ctrl, were considered either growth restricted (2 SDs below mean of means) or growth enhanced (2 SDs above mean of means).

### Tissue Collection

Tissue collection was done as previously described [12]. Briefly, following sacrifice of F1 female mice at 18.5 dpc, placenta and brain frontal cortices (anterior 1 mm of whole brains) from embryos were isolated, flash frozen and kept at −80°C until use.

### DNA Methylation Analyses

Analysis of imprinted gene DNA methylation was performed as previously described [12]. Frozen tissues were homogenized using mortar and pestle. DNA was isolated from ∼10mg of samples using the DNeasy Blood and Tissue kit (Qiagen, Germany). Bisulphite treatment of genomic DNA was performed using the EpiTect Bisulfite kit (Qiagen, Germany). Imprinted germline differentially methylated domains (gDMDs) were amplified using primers specific to pyrosequencing applications and sequenced using the PyroMark Gold Q24 Reagents (Qiagen) and the PyroMark Q24 Vacuum Workstation (Qiagen). Previously established primers used for assessment of *H19* [31], *Snrpn* [31], *Kcnq1ot1* [33], *Peg1* [34] and *Peg3* [33] are listed in Supplementary material and Table S1.

Global DNA methylation was assessed using the Luminometric Methylation Assay (LUMA) as previously described [12]. Briefly, duplicate digestions were carried out for both restriction enzymes HpaII and MspI (Thermo Fisher Scientific, USA). Samples were analysed on a Pyrosequencer Q24. Percentage methylation was calculated using the normalized peak ratios of HpaII over MspI as in the following formula: % Methylation = 100 [1-(HpaII/EcoRI/MspI/EcoRI)].

Reduced representation bisulphite sequencing (RRBS) libraries were generated using previously used and published protocols and the gel-free technique [35–37]. RRBS was carried out on *n* = 5–6 samples/diet/tissue (placenta and brain cortex). DNA samples of 500 ng were digested using the methylation-sensitive restriction enzyme, MspI, followed by end repair and A-tailing. Fragment size selection was performed with AMPure XP beads (Beckman Coulter, Brea, CA, USA). After ligation to methylated adapters (Illumina), DNA samples were bisulphite converted twice followed by a subsequent AMPure bead clean-up. RRBS libraries were prepared by large scale PCR. Placenta and brain cortex DNA libraries were then multiplexed for paired-end sequencing with twelve samples per lane of a HiSeq 2000 sequencer (Illumina) followed by initial data processing and alignment of reads by the software pipeline BSMAP (version 2.6) [38]. MethylKit software (version 0.5.3) was used for identification of differentially methylated tiles (DMTs) after folate deficiency or folic acid supplementation. This software implements the Benjamini–Hochberg false discovery (FDR)-based method for *P*-value correction and only DMTs passing the q-value threshold (*q *= 0.01) were considered [39]. Analysis was based on 100-bp step-wise tiling windows, containing ≥2 CpGs per tile and ≥10 × CpG coverage of each tile per sample. The methylation level of a 100-bp tile was the average of all CpGs within the tile. If significant changes of DNA methylation after folate deficiency or folic acid supplementation exceeded 10%, the tile was designated as a DMT; further annotation of DMTs was performed by the HOMER software (version 3.51). Summary statistics for RRBS are shown in Supplementary Table S2. DAVID Bioinformatic Functional Annotation Tool (v6.7) was used for gene ontology analysis.

As done previously, validation of RRBS results was carried by pyrosequencing on a subset of DMTs in placental samples [31, 37]. Details of the individual pyrosequencing assays are shown in Supplementary Table S3.

We also determined whether any DMTs were found in regions that would normally inherit DNA methylation marks from the maternal allele but not the paternal allele. To do this, we used publicly available methylation data from mouse sperm (GSE34864)[36], germinal vesicle oocytes (GVO-DRA00570) [40] and from the maternal allele from the inner cell mass (ICM) (GSE56697) [41]. Methylation data were obtained from each germ cell type/developmental stage and processed to obtain DNA methylation across 100-bp regions, similar to RRBS. To target regions where DNA methylation was specifically inherited from the maternal allele, we included only regions where sperm methylation was low (≤10%) and GVO/ICM methylation was greater than or equal 25%; these regions were then intersected with our DMTs using the intersectBed function of bedtools (version 2.26.0).

### Statistical Analysis

Results are expressed as the mean ± SEM, unless stated otherwise. Data were graphed and analysed with Prism 5 (GraphPad Software Incorporated, USA). Comparisons and statistical calculations were made by Fisher’s exact test (embryonic outcomes), ANOVA followed by the Dunnett’s multiple comparison test compared to control (reproductive outcomes), one-way ANOVA (global and imprinted gene methylation) or t-tests (sex differences in methylation). A level of significance for the analyses was set at *P* < 0.05.

## Results

### F1 Female Body Weight was Not Affected by Gestational and Early Postnatal FD and FS Diet Exposures

First, we evaluated the impact of the diets on outcomes in the F1 progeny and on the F1 female body weight as a general measure of health. The impact of the diets on F1 litter sizes has been reported previously; there were no significant differences observed in litter sizes or sex ratios between the diet and control groups [12]. Adult body weight did not differ between females exposed to control diets as compared to females exposed to the different FD and FS diets administered prenatally from conception to birth until weaning (Supplementary Fig. S1; mean 28.7, 28.4, 28.0 and 26.5 g in Ctrl, 7FD, 10FS and 20FS, respectively). Thus, as previously shown for F1 males [12], there was no apparent major effect of the diets on the health of the F1 females.

### Pre-Weaning Folate Deficiency or Folic Acid Supplementation during F1 Mothers’ Early Life Resulted in Decreased Offspring (F2) Litter Sizes and Increased Abnormal Outcomes

**Figure 2.**
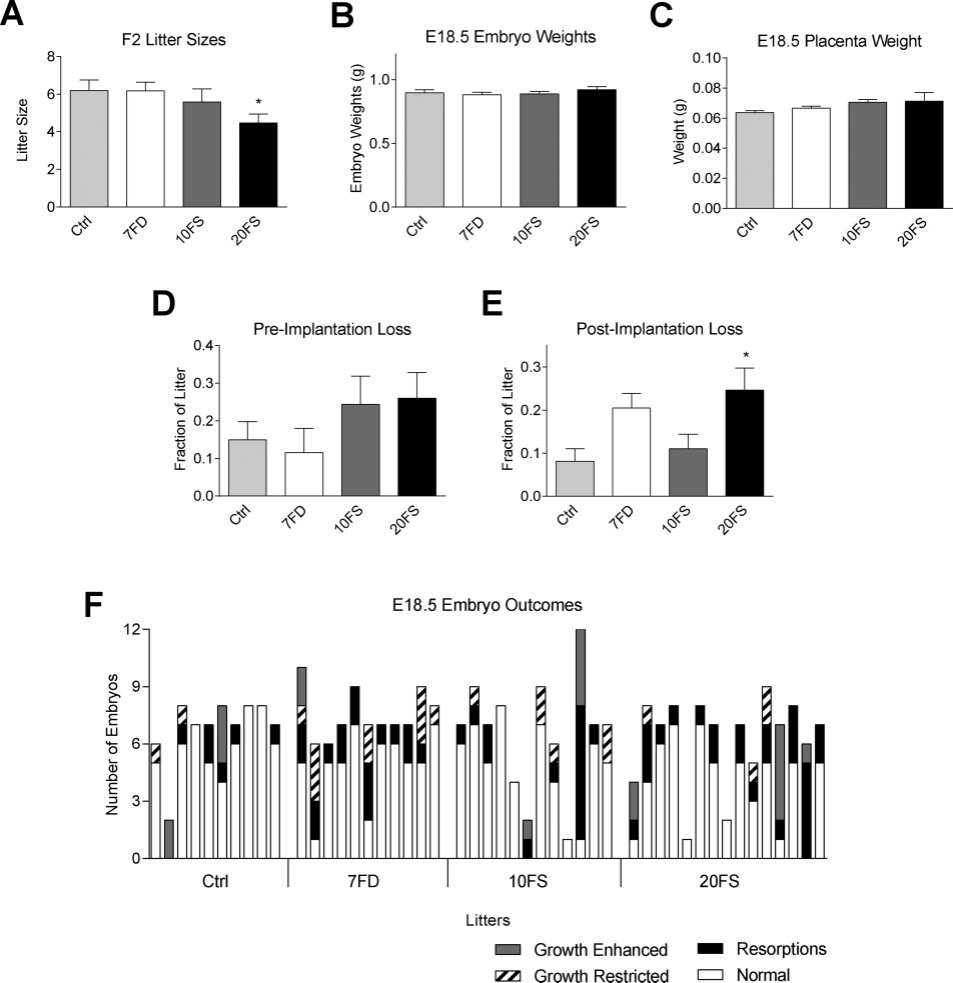
Effects of mothers' preweaning exposure to folate deficiency and folic acid supplementation on future reproductive outcomes in F2 at E18.5. (A) F2 litter sizes at E18.5 (*n* = 10–15 F2 litters). (B) F2 embryo weights at E18.5 and (C) F2 placental weights at embryonic day 18.5 (*n* = 49–59 embryos). (D) Pre-implantation loss of F2 at E18.5 (*n* = 10–15 F2 litters). (E) Post-implantation loss of F2 at E18.5. (F) Incidence of fetal abnormalities at embryonic day 18.5 per litter (*n* = 10–15 litters); growth restriction and enhancement are defined as a 2-fold SD difference of embryo weight to the group mean of litter mean weights (Ctrl, folic acid control diet; 7FD, 7-fold folic acid deficient; 10FS, 10-fold folic acid supplemented; 20FS, 20-fold folic acid supplemented). *P <0.05 by one-way ANOVA with Dunnett's multiple comparisons test.

Next, we assessed whether the oocytes of the F1 mothers had been impacted by the folate diets by examining effects on offspring of the F2 generation. F2 litters produced from F1 mothers from 20FS exposed groups displayed decreased litter sizes at E18.5 (Fig. 2A). F2 pre-implantation loss, and embryo and placenta weights were not affected by the mothers’ diet (Fig. 2B–D). However, increased resorptions amongst F2 litters at E18.5 from 20FS exposed F1 females were observed (Fig. 2E). The data shown in Fig. 2A–E are also shown as individual data points in Supplementary Fig. S2 with evidence of a few outliers in all groups.

Resorptions, growth enhancement or growth restriction were designated as abnormal outcomes. A trend was noted for a higher proportion of embryonic abnormalities among litters coming from F1 mothers exposed to the FD and FS diets as compared to mothers on the control diet (Fig. 2F). Whereas 4 of 10 control group litters had more than one abnormal outcome per litter, there were 24 out of 34 litters amongst the 7FD, 10FS and 20FS groups with more than one abnormal outcome per litter (*P*  = 0.08, Fisher’s exact test). Together the results suggest that the diets, in particular the 20FS, adversely affected F2 pregnancy outcomes. However, placenta weights were unaffected and those offspring that survived had normal weights at E18.5. Embryo/placental ratios are shown in Supplementary Fig. 2F, with no evidence of diet-associated altered ratios that would be indicative of placental insufficiency.

### Global and Imprinted Gene DNA Methylation in Offspring was Not Affected by F1 Mothers’ Early Life Exposure to Folate Deficiency or Folic Acid Supplementation

**Figure 3.**
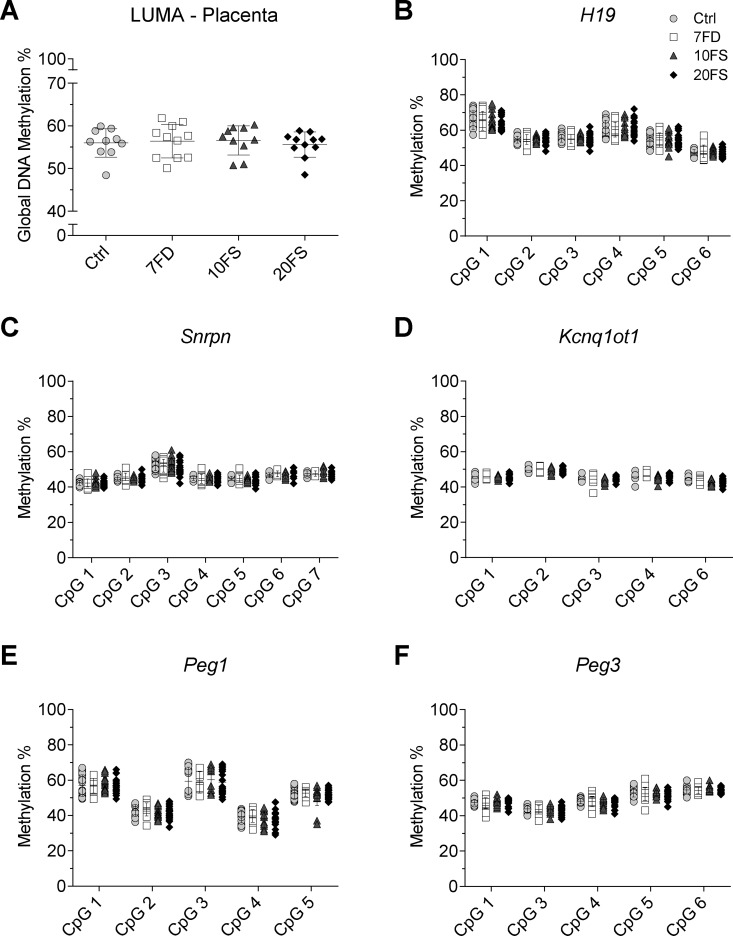
F2 E18.5 placenta global DNA methylation and DMR methylation at imprinted genes. (A) Global DNA methylation was measured using LUMA (*n* = 5–6/group/sex). Loci of paternally methylated gene (B) *H19* (*n* = 9–11/group/sex) and maternally methylated genes (C) *Snrpn*, (D) *Kcnq1ot1*, (E) *Peg1* and (F) *Peg3* (*n* = 9–11/group/sex) methylation levels were quantified by bisulphite pyrosequencing (Ctrl, folic acid control diet; 7FD, 7-fold folic acid deficient; 10FS, 10-fold folic acid supplemented; 20FS, 20-fold folic acid supplemented).

**Figure 4.**
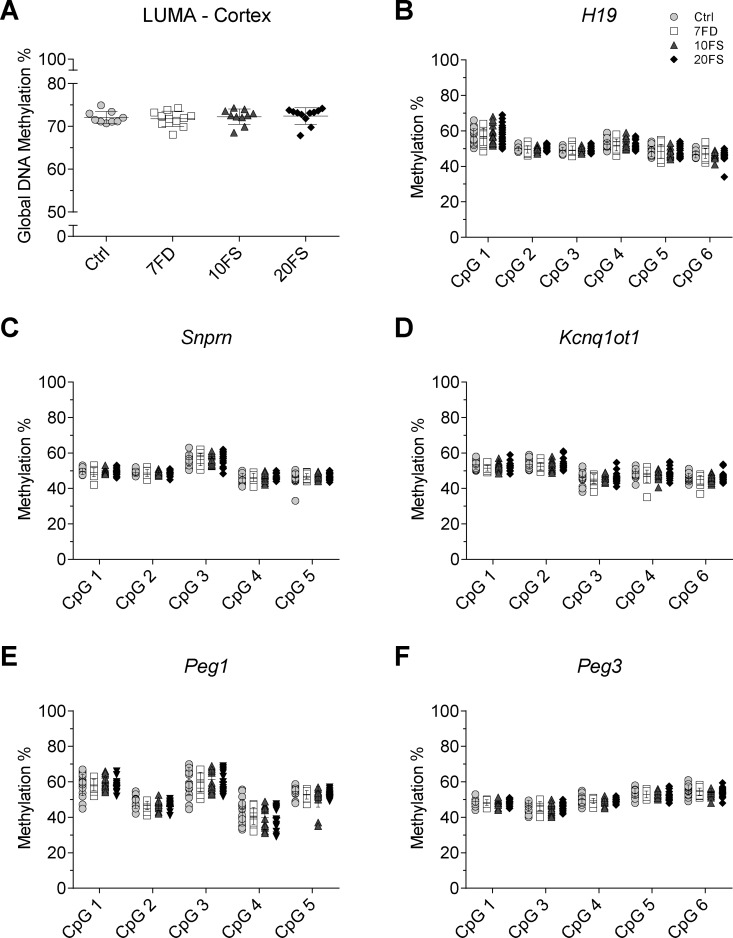
F2 E18.5 cortex global DNA methylation and DMR methylation at imprinted genes. (A) Global DNA methylation was measured using LUMA (*n* = 5–6/group/sex). Loci of paternally methylated gene (B) *H19* (*n* = 9–11/group/sex) and maternally methylated genes (C) *Snrpn*, (D) *Kcnq1ot1*, (E) *Peg1* and (F) *Peg3* (*n* = 9–11/group/sex) methylation levels were quantified by bisulphite pyrosequencing (Ctrl, folic acid control diet; 7FD, 7-fold folic acid deficient; 10FS, 10-fold folic acid supplemented; 20FS, 20-fold folic acid supplemented).

To determine if the decreased litter sizes and increased abnormal outcomes in F2 litters at E18.5 could be associated with epigenetic disturbances in the F1 oocytes that were inherited by the offspring, global and imprinted gene DNA methylation were determined in F2 E18.5 placentas (Fig. 3) and embryonic brain cortex (Fig. 4). E18.5 F2 embryos were chosen to represent offspring from F1 mothers of 10 different original F0 females per group. No evidence of sex differences was found in global methylation by LUMA (*n* = 5–6/group/sex) or in imprinted gene methylation (*n* = 9–11/sex/group) in either placental or cortex tissues (Supplementary Figs. S3 and S4, respectively). Therefore, both males and females were combined for downstream analysis.

Mean global methylation in placenta and brain cortex ranged from ∼55% to 73%, with no significant differences observed between the groups (Figs. 3A and 4A). An assessment was performed at a gene-specific level in placenta and brain cortex for the well characterized gDMDs of the paternally methylated imprinted gene *H19* and the maternally methylated imprinted genes *Snrpn*, *Kcnq1ot1*, *Peg1* and *Peg3*. Methylation levels averaging ∼50% for all imprinted genes were observed, as expected for a somatic or non-germ cell tissue (Figs 3B–F and 4B–F). Both global and imprinted gene methylation were assessed in males and females independently, and also showed no effect of the diets (Supplementary Figs. S3 and S4). Overall, no differences were observed for global and mean imprinted gene DNA methylation of placenta or cortex tissues.

### F1 Mothers’ Early Life FD and FS Diets are Associated with Subtle Genome-Wide DNA Methylation Alterations in the Placenta and Brain Cortex of F2 Offspring

To assess whether more subtle changes in genome-wide DNA methylation may have occurred in the F2 offspring, RRBS was performed on the placenta and brain cortex tissues at E18.5. RRBS was carried out on matched placenta and cortex sample of males (*n* = 5–6/tissue/group) as no evidence of sex differences had been found for the methylation of imprinted genes. Analysis of ∼1.5 million CpGs was performed in 100-bp tiles to identify regions with differential methylation between the low or high folate and control groups (i.e.100-bp tiles exhibiting gain or loss of 10% methylation from the FD and FS exposures: DMTs). Heatmaps of the RRBS results are shown in Supplementary Fig. S5. For all samples, as well the individual groups, the cortex and placenta samples clearly clustered within the individual tissue type. For the data as a whole, within placenta or cortex, there was no evidence of clustering between groups suggesting that overall genome-wide methylation differences between the groups were small, requiring a more detailed analysis of individual tiles using MethylKit.

**Figure 5.**
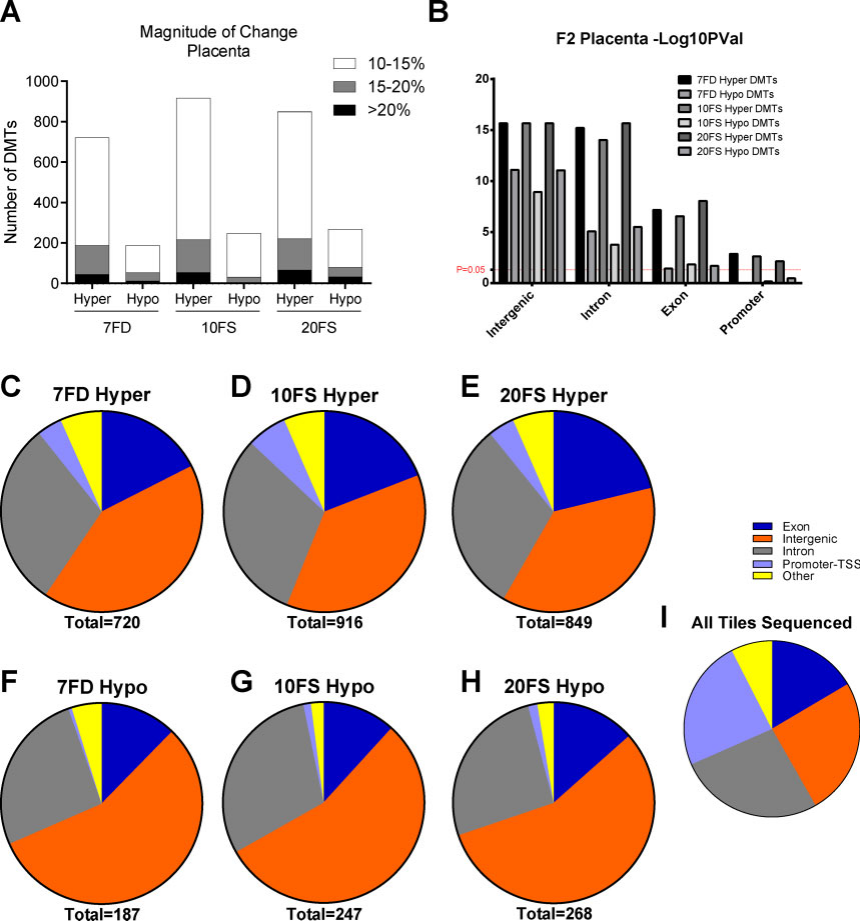
Distribution of DMTs in placenta by genomic regions following perinatal maternal FD and FS exposure. (A) Magnitude of methylation change in DMTs in post treatment groups. (B) Statistical significance [−log(*P-*val)] of difference in methylation within genomic region DMTs compared to Ctrl. (C–H) Prevalence of genomic regions affected within DMTs. (I) Genomic region distribution among all tiles covered and sequenced by RRBS (*n* = 6 males/group; 7FD, 7-fold folic acid deficient; 10FS, 10-fold folic acid supplemented; 20FS, 20-fold folic acid supplemented).

Using MethylKit, the F1 mothers’ early life exposures to the 7FD, 10FS and 20FS diets resulted in 907, 1163 and 1116 DMTs, respectively, in placentas of F2 offspring (Fig. 5). Most alterations were of low magnitude of 10–15% (Fig. 5A). However, some DMTs displayed higher magnitude (15–20%) changes in methylation (184/907, 191/1163 and 202/1116 in 7FD, 10FS and 20FS exposure groups, respectively), while considerably less DMTs displayed a greater than 20% change (56/907, 56/1163 and 96/1116 in 7FD, 10FS and 20FS exposure groups, respectively). Regardless of the diet group, the majority of the DMTs showed a gain of methylation, with a range of 76–79% hypermethylated DMTs (Fig. 5A and Supplementary Table S4). We observed a significant enrichment of intergenic regions in DMTs following both FD and FS perinatal maternal exposures compared to all tiles sequenced (average of 47% with range of 37–56% for diet-associated intergenic DMTs vs 25%, all tiles sequenced that were intergenic; Fig. 5B and C–I and Supplementary Table S5).

**Figure 6.**
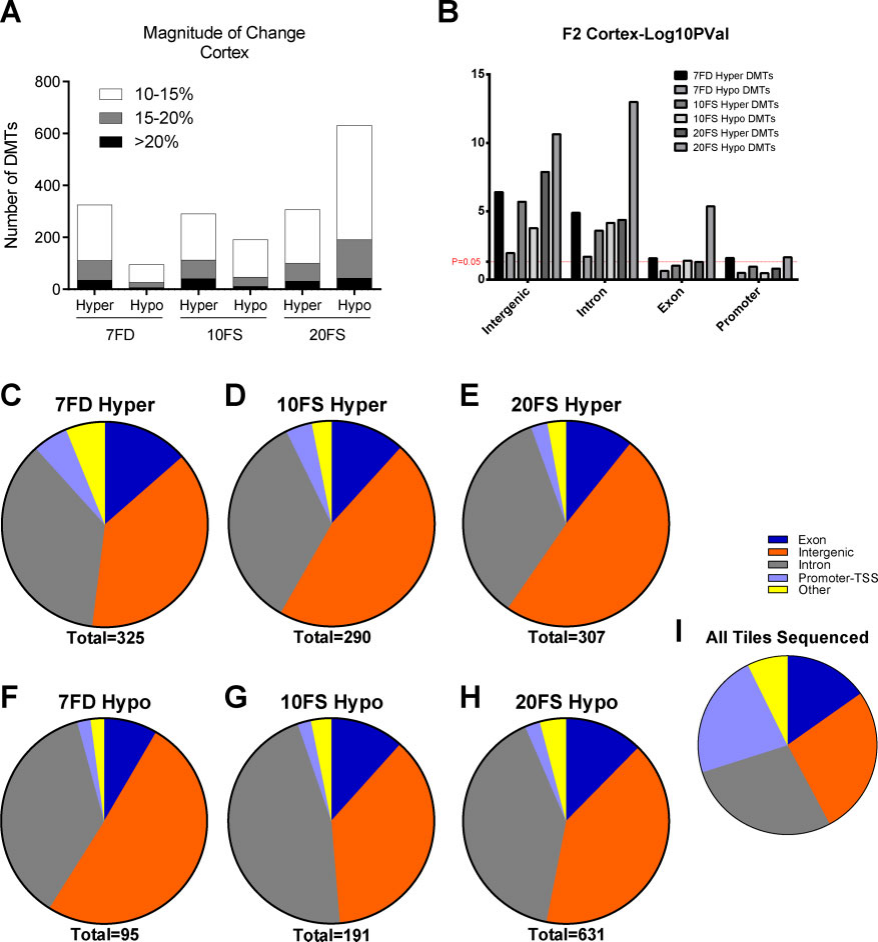
Distribution of DMTs in cortex by genomic regions following perinatal maternal FD and FS exposure. (A) Magnitude of methylation change in DMTs in post treatment groups. (B) Statistical significance [−log(*P-*val)] of difference in methylation within genomic region DMTs compared to Ctrl. (C–H) Prevalence of genomic regions affected within DMTs. (I) Genomic region distribution among all tiles covered and sequenced by RRBS. (*n* = 5–6 males/group; 7FD, 7-fold folic acid deficient; 10FS, 10-fold folic acid supplemented; 20FS, 20-fold folic acid supplemented).

RRBS was also performed on placenta-matched frontal cortex of E18.5 F2 embryos and DNA methylation of FD and FS groups was compared to Ctrl (Fig. 6). Perinatal maternal exposures to 7FD, 10FS and 20FS diets resulted in 421, 481 and 926 DMTs, respectively, in F2 offspring frontal cortices, with the magnitude of change mostly in the 10–15% range (Fig. 6A). Fewer DMTs displayed a higher magnitude (15–20%) change in methylation (75/421, 107/481 and 218/926 in 7FD, 10FS and 20FS exposure groups, respectively), while even less DMTs displayed changes of 20% or greater (35/421, 51/481 and 64/926 in 7FD, 10FS and 20FS exposure groups respectively). Similar to the placenta, both FD and 10FS exposures resulted in a higher prevalence for gain of methylation with 60–77% of DMTs hypermethylated for the cortex (Fig. 6A and Supplementary Table S4). In contrast, 20FS exposures in the F1 mothers’ early life resulted in a greater number of hypomethylated versus hypermethylated DMTs (68% vs 32%, respectively). Again, for all groups, DMTs were significantly enriched in intergenic regions (average of 44% for diet-associated intergenic DMTs vs 27% all tiles sequenced that were intergenic; Fig. 6B–I and Supplementary Table S6).

DNA methylation for a subset of affected loci (hypomethylated and hypermethylated sites) identified by RRBS for the placenta was examined using a second assay, bisulphite pyrosequencing, in all cases validating the RRBS results (Supplementary Table S7).

### Genes and Pathways Associated with DNA Methylation Alterations in F2 Offspring

DNA methylation alterations associated with nearby genes (i.e. genic DMTs) were assessed to determine potential functional impacts on the placenta and cortex. In F2 placentas, all three diets resulted in genic region-associated DMTs with statistically significant enrichment of various pathways. Notably, pathways implicated in transcription, multicellular organism development and nervous system development were commonly affected in all three exposures (Table 1). Two pathways consistently had the greatest number of differentially methylated genes, with 26 genes implicated in multicellular organism development and 25 genes involved in transcription and common to all three diet exposures (Supplementary Table S8).

**Table 1.** DAVID bioinformatics analysis showing all the significantly enriched biological pathways for all genic DMTs in the F2 placenta, for each exposure group

Tissue	Pathway	No. of genes	Benjamini
7FD DMTs			

Placenta	Multicellular organism development	53	6.97E−05
	Transcription, DNA templated	71	4.91E−04
	Regulation of transcription from RNAPII promoter	44	1.26E−03
	Angiogenesis	17	7.08E−03
	Nervous system development	21	1.63E−02

10FS DMTs			

Placenta	Multicellular organism development	67	2.76E−09
	Transcription, DNA-templated	87	2.04E−05
	Regulation of transcription from RNA polymerase II promoter	52	4.52E−04
	Epithelial cell differentiation	11	2.64E−03
	Nervous system development	25	1.05E−02
	Establishment of planar polarity	6	1.99E−02
	Anterior/posterior pattern specification	12	2.77E−02
	Regulation of ion transmembrane transport	13	2.92E−02
	Cochlea morphogenesis	6	4.06E−02
	Planar cell polarity pathway involved in neural tube closure	5	3.92E−02
	Axon guidance	13	5.69E−02

20FS DMTs			

Placenta	Multicellular organism development	71	3.73E−11
	Regulation of transcription from RNAPII promoter	54	1.93E−04
	Transcription, DNA templated	84	1.52E−04
	Nervous system development	25	1.39E−02
	Transmembrane receptor protein tyrosine kinase signaling pathway	11	3.29E−02

In the cortex, when only genic DMTs were considered, the top five pathways are listed in Table 2. Only one significantly enriched pathway was identified after correction (substrate adhesion-dependent cell spreading; 8 genes) for the 7FD group.

**Table 2.** DAVID bioinformatics analysis showing all the significantly enriched biological pathways for all genic DMTs in the F2 cortex, for each exposure group

Tissue	Pathway	No. of genes	*P*-value	Benjamini
7FD DMTs				

Cortex	Substrate adhesion-dependent cell spreading	8	2.08E−05	4.19E−02
	Protein phosphorylation	28	1.43E−04	2.55E−01
	Positive regulation of gene expression	22	1.66E−04	2.90E−01
	Cellular response to retinoic acid	8	4.88E−04	6.34E−01
	Regulation of neurotransmitter secretion	5	7.85E−04	8.02E−01

10FS DMTs				

Cortex	Cytoskeleton organization	7	2.81E−03	9.76E−01
	Transcription, DNA-templated	40	4.21E−03	9.40E−01
	Ovarian follicle development	5	7.20E−03	9.60E−01
	Multicellular organism development	24	1.17E−02	9.80E−01
	Regulation of alternative mRNA splicing, via spliceosome	4	1.53E−02	9.84E−01

20FS DMTs				

Cortex	Protein phosphorylation	33	3.09E−05	6.26E−02
	Phosphorylation	32	2.17E−04	2.03E−01
	Protein autophosphorylation	14	8.25E−04	4.37E−01
	Synapse organization	6	2.30E−03	7.00E−01
	*Wnt* signaling pathway	14	3.20E−03	7.38E−01

We intersected the DMT lists from the placenta and the cortex tissues to identify regions that had DNA methylation simultaneously altered in both tissue types. Of the DMTs found within genic regions 46, 55 and 71 were conserved across both placenta and cortex tissue types within the 7FD, 10FS and 20FS groups, respectively (Supplementary Table S9). Of the tissue-conserved DMTs in the 7FD group, 45 of 46 showed the same directionality of methylation change in both tissues; similar directionality was seen for 49 of 55 loci for the 10FS exposure and 51 of 71 loci for the 20FS group. When considering tissue-conserved DMTs with the following two criteria: same directionality of effect and at least one of the two tissues having greater than a 20% change in methylation, there were 11, 9 and 15 DMTs meeting these criteria in the 7D, 10FS and 20FS groups, respectively. A number of genes were affected in more than one diet group: *Map1lc3b* (hypomethylated), *Arhgef26* (hypermethylated) and *Ckb* (hypermethylated) in the three groups, *Olfr136* (hypermethylated) and *Mir153* (hypermethylated) in the 7FD and 20FS groups and *Rab11fip3* in the 10FS and 20FS groups.

**Table 3.** DMTs in placenta which intersect with methylation data from GVO and ICM studies by Shirane *et al*. [40] Wang *et al*. [41]

Methylation change from GVO→ICM	Number of placenta DMTs								
	7FD			10FS			20FS		
	Hyper	Hypo	All DMTs	Hyper	Hypo	All DMTs	Hyper	Hypo	All DMTs
Gain	7	4	11	6	1	7	5	4	9
Stable	196	37	233	269	46	315	222	61	283
Loss	133	33	166	167	38	205	170	41	211
Total	336	74	410	442	85	527	397	106	503

Distribution of DMTs is done among regions that undergo *de novo* DNA methylation (gain) or DNA methylation erasure (loss) on the maternal allele during the transition from germinal vesicle stage oocyte to the ICM (7FD, 7-fold folic acid deficient; 10FS, 10-fold folic acid supplemented; 20FS, 20-fold folic acid supplemented; Hyper, hypermethylated DMTs; Hypo, hypomethylated DMT; Gain and loss of methylation was defined as an increase or decline in methylation of ≥10% from GVO to ICM).

**Table 4.** DMTs in cortex which intersect with methylation data from GVO and ICM studies by Shirane *et al*. [40] and Wang *et al*. [41]

Methylation change from GVO→ICM	Number of cortex DMTs								
	7FD			10FS			20FS		
	Hyper	Hypo	All DMTs	Hyper	Hypo	All DMTs	Hyper	Hypo	All DMTs
Gain	4	1	5	3	0	3	3	5	8
Stable	81	26	107	82	30	112	82	135	217
Loss	63	9	72	56	39	95	53	128	181
Total	144	35	179	138	69	207	135	263	398

Distribution of DMTs is done among regions that undergo *de novo* DNA methylation (gain) or DNA methylation erasure (loss) on the maternal allele during the transition from germinal vesicle stage oocyte to the ICM (7FD, 7-fold folic acid deficient; 10FS, 10-fold folic acid supplemented; 20FS, 20-fold folic acid supplemented; Hyper, hypermethylated DMTs; Hypo, hypomethylated DMT; Gain and loss of methylation was defined as an increase or decline in methylation of ≥10% from GVO to ICM).

### DMTs Coincide with Loci Retaining Methylation on the Maternal Allele during Post-Fertilization Development

Next we wanted to assess whether the folate diets had affected oocyte loci of potential significance for the health of the offspring of the next generation. A number of loci, including the maternally methylated differentially methylated regions (DMRs) of imprinted genes show high levels of methylation in oocytes and this methylation is maintained during the demethylation wave that occurs during pre-implantation development. For such maternally methylated loci, that also have low methylation on the paternal allele, offspring thus inherit methylation from their mothers and it may have functional significance in the progeny. For instance, in the case of imprinted genes, failure to inherit the normal maternal methylation patterns impacts growth and development of the offspring. Here we determined whether any DMTs were found at loci that normally inherit methylation from the maternal allele. Previously published datasets that characterized genome-wide methylation states in GVO and the ICM were analysed in order to identify DMTs in loci for which methylation patterns have been shown to be inherited from the oocyte, resisting demethylation in pre-implantation embryos [40, 42, 43]. For the ICM we restricted our analysis to the maternal allele (ICMm). We cross-referenced our DMTs to regions associated with low methylation in sperm (≤10%) but moderate to high methylation in GVO and ICMm (≥25%) for placenta and cortex (Table 3 and 4, respectively). For placenta, there were 50, 67 and 55 DMTs, and for cortex there were 19, 22 and 37 DMTs, representing the 7FD, 10FS and 20FS groups, respectively, localized to regions methylated in GVO and ICMm (Supplementary Table S10). For each tissue the specific DMTs identified are listed along with the associated genes, the degree of alteration in DNA methylation and the level of methylation of the loci taken from the published GVO and ICMm data (Supplementary Table S11). Notably, the majority of DMTs in each group and tissue were characterized as having levels of methylation ≥75% in GVO.

Among the placenta DMTs identified, the majority were hypermethylated (80%, 94% and 80%) and were predominantly in genic regions (77–100%; Supplementary Tables S10A and S11). For the cortex, while most identified DMTs in the 7FD and 10FS groups were hypermethylated (84% and 77%, respectively), the majority of DMTs in the 20FS diet group were hypomethylated (65%). Cortex DMTs were also predominantly in genic regions (66.7–87.55%; Supplementary Table S10B). Most of the groups for each tissue had one DMT localized to an imprinted (*Wars* within the *Dlk1-Gtl2* region, *Snurf*, *Peg 13*, *Plagl*) or imprinted-like region (*L3mbt11*) [35]. The DMTs identified for both tissues were enriched in pathways implicated in transcription, multicellular organism development and nervous system development. However, no statistical significance of pathway enrichment remained upon correction (Supplementary Table S12).

## Discussion

The effects of maternal folate deficient and folic acid supplemented diets during gestation and their direct consequences on offspring health have been well studied [44, 45]. Less is known about the impact of gestational exposures to low and high folate on the epigenome of developing germ cells, in particular those of females, and whether subsequent generations are affected. Using a mouse model, we demonstrated that an exposure to either folate deficiency or folic acid supplementation in females, including their developing germ cells, during early life can have adverse effects on their future reproductive health and progeny. Both reproductive loss and alterations in DNA methylation were found in offspring as a result of these exposures. Both the timing and doses were relevant to clinical folic acid use in high risk pregnancies. By weaning the F1 females onto control diets, our study design restricted the diet exposures of the F1 mothers to the *in utero* stages of female germ cell development along with the pre-weaning phase of perinatal life. Effects observed in the F2 offspring would thus be due to disruptions in the F1 oocytes resulting from exposures to low or high folate prior to the primordial follicle stage.

The effects of diet exposures on red blood cell (RBC) and plasma folate concentrations, as well as the reproductive outcomes of F0 females in this study, have been previously reported [12]. Briefly, when compared to the control diets, F0 dams that consumed a FD diet had ∼4× lower plasma and RBC folate concentrations, whereas mice consuming the supplemented diets had ∼2× higher plasma and RBC folate concentrations. In the early lifetime diet exposed F1 females, no differences in mean adult body weights were observed. Although the F1 females were exposed to their mothers’ low and high folate levels prenatally and up until weaning, as adults they were of normal weight and apparent health when they initiated their own pregnancies to produce the F2 generation.

In our previous study, we explored effects of male germ cell exposure to the same diets used here on offspring reproductive and epigenetic outcomes [12]. Due to its role in one-carbon metabolism we expected the low and high folate diets to perturb methyl donors needed for the dramatic reprogramming of DNA methylation that takes place in the germline. Before being mated to produce the F2 generation, F1 males were exposed to the diets during prenatal development, when DNA methylation in germ cells is erased across much of the genome, followed by reacquisition at most sites just before birth, as well as postnatally when DNA methylation patterns continue to be remodeled during spermatogenesis. In the male study, the F2 generation exhibited decreased litter sizes, fetal abnormalities and increased postnatal death that were highest in the 20FS group as compared to the 7FD and 10FS groups; all three groups showed evidence of epigenetic perturbations in imprinted genes. As similar reproductive and epigenetic effects were seen in the three diet groups, we proposed that the 10FS and 20FS diets resulted in the equivalent of a folate deficient state due to down regulation of folate metabolism pathway enzymes. In support of this, we and others have shown that high doses of folic acid in the 10FS to 20FS range can downregulate enzymes such as MTHFR in the one-carbon metabolism pathway, paradoxically leading to a decrease in the availability of SAM for cellular methylation reactions [30, 46].

In this study, female germ cells in the F1 mothers were only exposed to the folate diets during the DNA methylation erasure but not the remethylation phase in female germ cells. We thus postulated that effects on reproductive and epigenetic outcomes in the F2 might be less severe than those seen in the male study. Contrary to our expectations, an increase in resorptions and decrease in litter size were found in the 20FS group. Post-implantation loss was higher than control in the 7FD group but did not reach significance. As a caveat, we cannot rule out the possibility that uterine defects in the F1 females may have contributed to the resorptions; however, we consider this interpretation unlikely due to the apparent normal general health of the F1 females. A trend to increased abnormal outcomes (growth restriction, growth enhancement and resorptions) was observed across all three diet exposure groups. These results gave us the first indication that the folate diets had adversely affected the oocytes of the F1 mothers.

To better understand the mechanisms underlying the reproductive effects of the folate diets, as a measure of potentially heritable epigenetic effects of the exposures, we next examined DNA methylation patterns in the offspring. Two tissues in the F2 offspring were chosen for study. The placenta was chosen as it often shows higher levels of DNA methylation defects associated with perturbation of peri-conceptional epigenetic reprogramming events than do somatic tissues in the fetus [47, 48]. The brain cortex was chosen as a representative embryonic somatic tissue to examine. Assessment of global DNA methylation by LUMA showed no difference in the mean levels of methylation in placenta or cortex among the diet groups. The LUMA results indicated that large-scale changes in DNA methylation were not present in viable fetuses as a result of maternal folate exposures, a similar finding to that of the male study with the same diets.

The imprint control regions (ICRs) or gDMDs of imprinted genes are reprogrammed in primordial germ cells in order to take on maternal- or paternal-specific methylation patterns that dictate allele-specific expression in offspring. Thus, we examined the methylation of the well characterized gDMDs of several maternally and paternally methylated imprinted genes and did not reveal any perturbations in the placenta or cortex of male or female offspring. These results contrast with those of the male folate diet experiments where F2 offspring showed variation in methylation of the same gDMDs of imprinted genes examined in this study, suggesting the induction of epigenetic instability at these loci in developing male germ cells. We suggest that imprinted genes may have been more affected in the male experiment as germ cells were exposed to the low and high folate diets during both DNA demethylation and remethylation. In contrast, in the female experiment described in this paper, germ cells were only exposed during the DNA demethylation phase of germ cell development.

Uncovering subtle effects at developmentally important sequences that might help explain the abnormal pregnancy outcomes requires higher resolution genome-wide approaches. Therefore, we performed RRBS. Large numbers of DMRs were found in all three diet groups for both tissues, with roughly twice as many regions affected in placenta as in brain cortex. When compared to Ctrl diets, with the exception of the 20FS cortex group, F2 placenta and cortex for all the other diet groups had more hypermethylated DMTs (60–79%) than hypomethylated DMTs (21–40%). The presence of predominantly hypermethylated DMTs in the offspring, despite *de novo* DNA methylation not occurring in the oocyte during the exposure window is intriguing. The fact that the folate deficient and supplemented diets showed remarkably similar effects, resulting in both hyper- and hypomethylation, supports the suggestion of a common underlying biochemical/molecular explanation.

The question thus arises as to how the folate diets resulted in DNA methylation defects in the F2 offspring when the diet exposures preceded the DNA remethylation phase in F1 oocytes. Recent high-resolution studies have indicated that histone modifications and transcriptional activity control the unique DNA methylation landscape of the oocyte genome (reviewed in [49]). DNA methylation in oocytes occurs for the most part in actively transcribed regions including gene bodies; in contrast, intergenic, transcriptionally inactive regions have low levels of DNA methylation. Dynamic changes in histone modifications precede DNA methylation in oocytes [22]. H3K36me3 marks regions of DNA methylation whereas sites of H3K4me3 are found in regions where DNA methylation levels are absent or low [23, 43, 50]. Thus, it is possible that altered methyl donor availability from the folate diet exposures could disrupt normal H3K4me3 and H3K36me3 dynamics and subsequently DNA methylation patterns. For instance, in the oocyte-specific SETD2 knockout, H3K36me3 depletion in oocytes results in a decrease in gene-body and imprinted gene ICR DNA methylation and the appearance of DNA methylation in regions of the genome that are normally unmethylated [23].

Interestingly, in contrast to all other comparisons, the F2 20FS brain cortex samples showed predominantly hypomethylated DMTs rather than hypermethylated DMTs. For the cortex, the 20FS group also had the highest number of DMTs compared to the 7FD and 10FS groups. Reproductive outcomes were also most marked in the 20FS group. Notably, the 20FS cortex DMTs were found in genic regions, in introns and exons. As transcription is important in setting DNA methylation patterns in female germ cells, it is possible that altered transcription in oocytes exposed to the 20FS dose could contribute to the hypomethylated genic DMTs. However, if altered transcription was occurring, it is unclear why the 20FS placenta samples did not show predominantly hypomethylated DMTs as well. Compared to other tissues, brain has particularly high levels of 5-hydroxymethylcytosine (5-hmC) [51]. As RRBS cannot distinguish between 5-methylcytosine (5-mC) and 5-hmC, an alternate possibility is that altered 5-hmC may also help explain the predominance of hypomethylated DMTs in the 20FS group cortex.

A full understanding of the molecular mechanisms underlying the effects of the folate diets on developing oocytes will require high-resolution studies of histone modifications and DNA methylation in isolated oocytes from the fetal and neonatal ovaries of the F1 females. In the meantime, more detailed examination of the DNA methylation perturbations in the placenta and cortex can shed light on the types of sequences affected and the DNA methylation defects that persist in association with folate diet effects on developing female germ cells. With our gene ontology analysis of the DMT lists, several interesting pathways and genes emerged. In the placenta, an enrichment in genes related to both multicellular organism development and transcription was found, regardless of exposure group. Disturbances in either pathway, but especially the multicellular organism development pathway, could explain or contribute to the increased resorptions and other developmental abnormalities. For instance, *Alx3* expression has been reported to prevent malformations and deficiencies are associated with NTDs and craniofacial abnormalities [52, 53]. Concurrently, another consistently affected gene *Angpt2*, has been linked to pre-eclampsia, general placental function and fetal health [54, 55]. Finally, transcription factors *Tbx1* and *Hoxa10* have both been shown to have wide reproductive and developmental roles, whilst having epigenetic functions and being under epigenetic regulation, respectively [56, 57].

Interestingly a minority of DMTs (7–11%) in the cortex corresponded to DMTs found in the placenta, suggesting that there are tissue-specific responses and susceptibilities to environmental exposures in the pre-conceptus oocyte. Additionally, no consistent biological pathways were enriched in the cortex DMTs. Genic DMTs conserved in both placenta and cortex frequently affected 1st introns, which have been shown to be highly conserved and play roles in expression regulation [58]. Although the effects of these epigenetic changes in tissues of the offspring and their developmental outcome are difficult to infer, transcriptional dysregulation remains a viable hypothesis. The majority of DMTs occurred in intergenic regions, areas known to house regulatory elements. Indeed, placental DMTs were found to have a ∼3.3- to 3.6-fold enrichment of placenta-specific regulatory elements in the 10FS and 20FS exposures.

Examination of loci known to inherit their DNA methylation patterns from the oocyte, but not the sperm allowed us to further probe the potential functional impact of the folate diets on DNA methylation in specific genomic regions. Parent-of-origin inherited methylation has important roles in embryonic growth and development; such marks have classically been shown to be critical in the case of imprinted genes. In our analysis, many interesting genes were identified and, although no pathways were significantly affected following correction, similar pathways to those emerging from the overall RRBS data analysis were identified. The finding that genic regions were predominantly affected by the diets suggests that regions where DNA methylation normally takes place in oocytes, targeted by H3K36me3 marks, may be particularly susceptible to the low and high folate exposure.

Together, our results indicate that low and high dietary folate exposure of oocytes in the time preceding reacquisition of DNA methylation can impact oocyte health, leading to altered reproductive and epigenetic outcomes. The mechanisms underlying the adverse reproductive outcomes will require further study by examining offspring at earlier stages of development and performing DNA methylation along with gene expression studies. The identification of DNA methylation defects in neurodevelopmental pathways suggests a potential target for study of offspring during postnatal development. This study tested the effects of low and high folate diets on early oocytes, an exposure relevant to the clinical use of folic acid supplements during pregnancy. In future studies, as women take folic acid supplements prior to becoming pregnant, it will be interesting to study the offspring and epigenetic impacts of exposure to the same folate diets during the window of DNA methylation acquisition in growing oocytes.

## Supplementary data

Supplementary data are available at *EnvEpig* online.

## Supplementary Material

dvaa018_Supplementary_Data
