# Supplementary material for: Impact of mothers' early life exposure to low or high folate on progeny outcome and DNA methylation patterns
Source: Environ Epigenet. 2020 Nov 18;6(1):dvaa018. doi: 10.1093/eep/dvaa018 (PMC7673481; doi:10.1093/eep/dvaa018)
Supplement: dvaa018_Supplementary_Data [file dvaa018_supplementary_data.pdf]

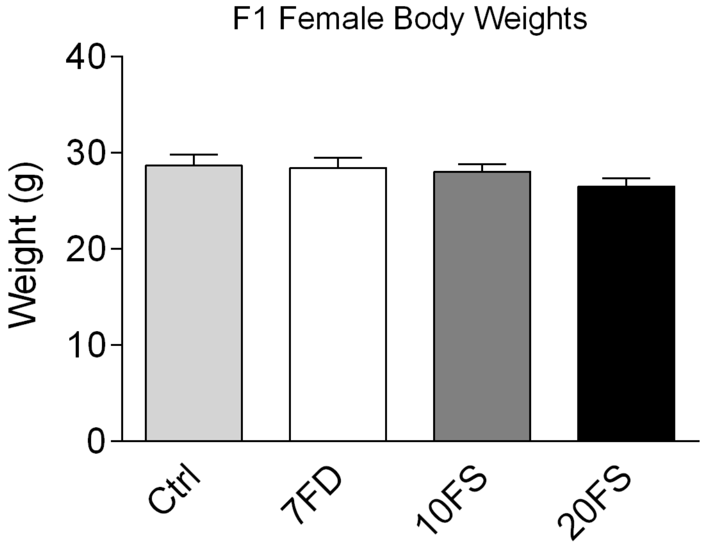

Supplementary Figure S1. Effect of prenatal folate deficiency and supplementation on F1 adult female body weight (n = 17-20/group) (Ctrl = Folic Acid Control Diet, 7FD = 7x Folic Acid Deficient, 10FS = 10x Folic Acid Supplemented, 20FS = 20x Folic Acid Supplemented). * =p<0.05 by one-way ANOVA with Dunnett's multiple comparisons test.

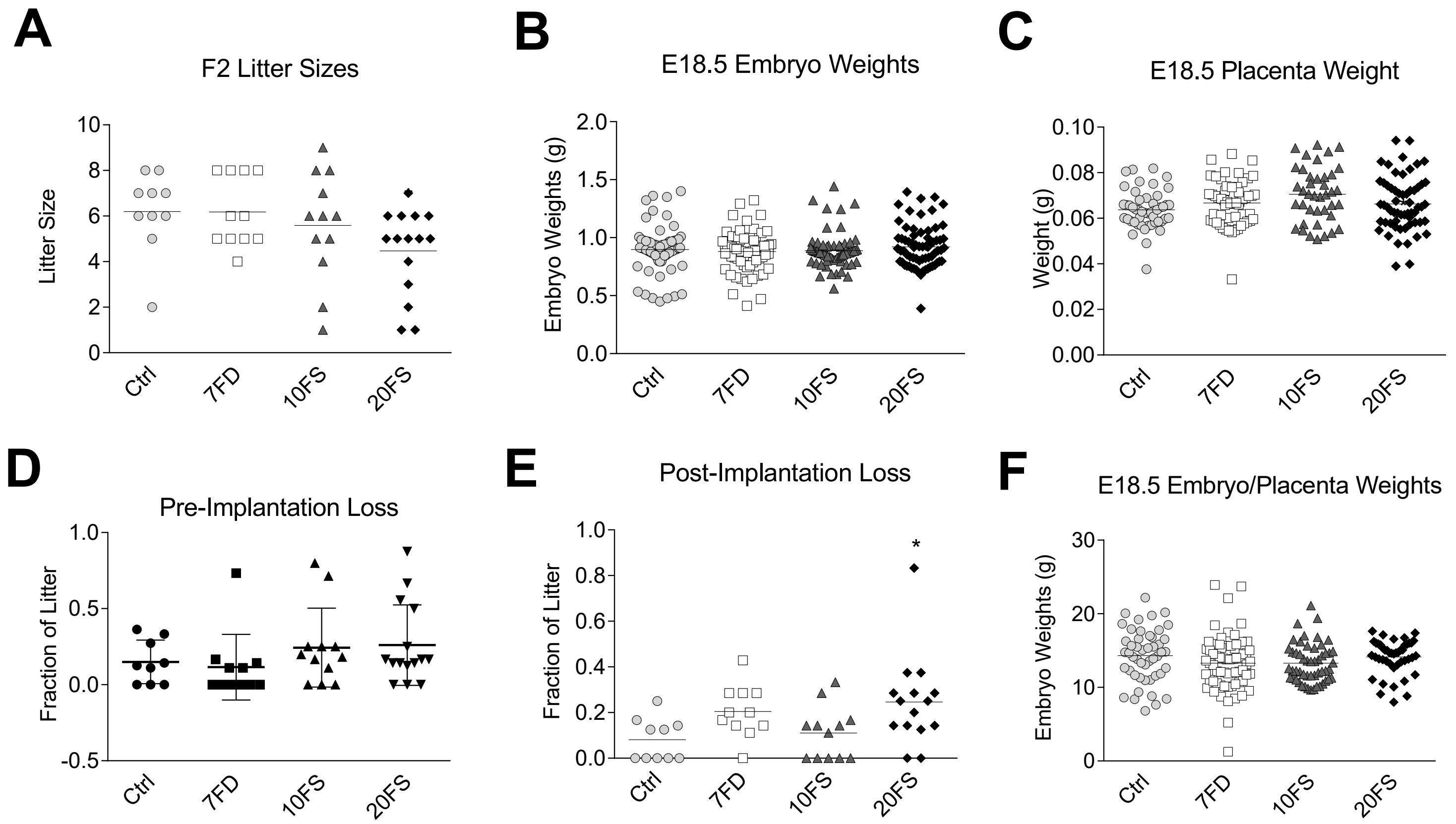

Supplementary Figure S2. Effects of mothers' preweaning exposure to folate deficiency and folic acid supplementation on future reproductive outcomes in F2 at E18.5. Individual data points were plotted for (A) F2 litter sizes at E18.5 (n = 10-15 F2 litters), (B) F2 embryo weights at E18.5, (C) F2 placental weights at embryonic day 18.5 (n = 49-59 embryos), D) pre-implantation loss of F2 at E18.5 (n = 10-15 F2 litters), (E) Post-implantation loss of F2 at E18.5 and (F) the ratio of embryo to placenta weight (n = 49-59). (Ctrl = Folic Acid Control Diet, 7FD = 7x Folic Acid Deficient, 10FS = 10x Folic Acid Supplemented, 20FS = 20x Folic Acid Supplemented). * = $p<0.05$ by one-way ANOVA with Dunnett's multiple comparisons test.

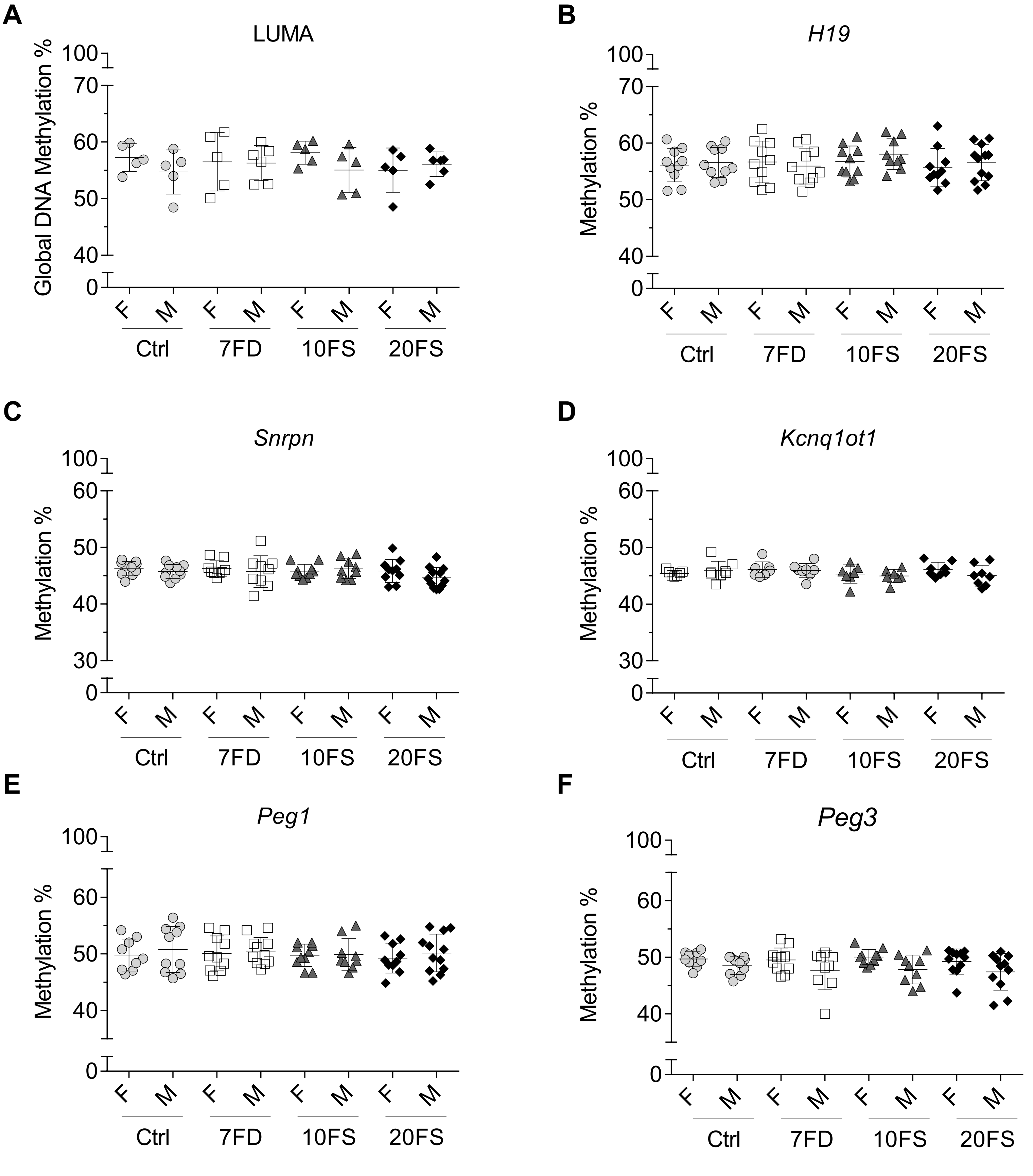

Supplementary Figure S3. Individual data points for F2 E18.5 placenta global DNA methylation and DMR methylation at imprinted genes. A) Global DNA methylation was measured using LUMA (n =5-6/ group/sex). Loci of paternally methylated gene B) *H19* (n =9-11/group/sex) and maternally methylated genes C) *Snrpn*, D) *Kcnq1ot1*, E) *Peg1* and F) *Peg3* (n = 9-11/group/sex) methylation levels were quantified by bisulfite pyrosequencing. (Ctrl = Folic Acid Control Diet, 7FD = 7x Folic Acid Deficient, 10FS = 10x Folic Acid Supplemented, 20FS = 20x Folic Acid Supplemented).

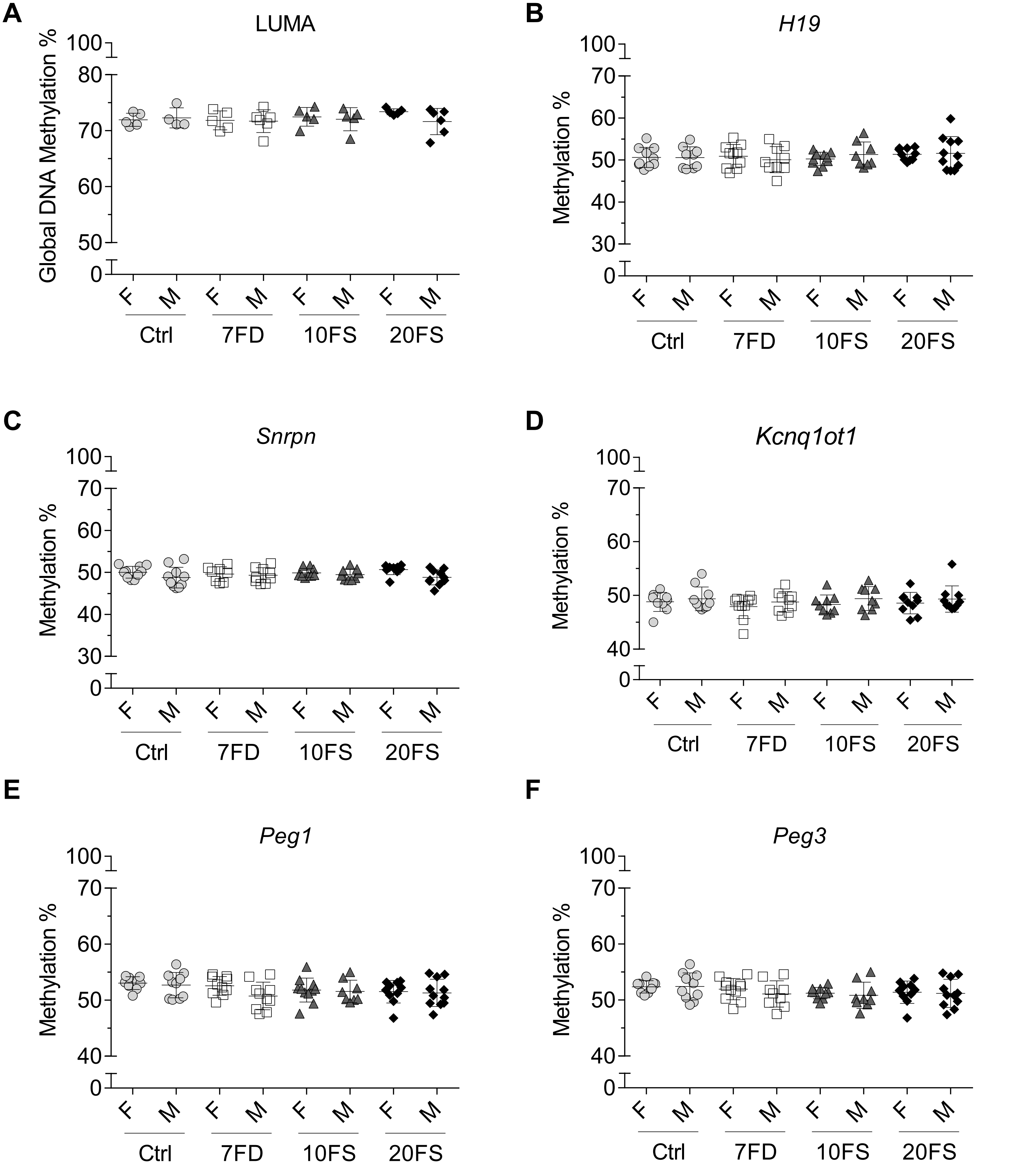

Supplementary Figure S4. Individual data points for F2 E18.5 cortex global DNA methylation and DMR methylation at imprinted genes. Global DNA methylation was measured using LUMA (n =5-6/group/sex). Loci of paternally methylated gene A) *H19* (n = 9-11group/sex) and maternally methylated genes B) *Snrpn*, C) *Kcnq1ot1*, D) *Peg1* and E) *Peg3* (n = 9-11/group/sex) methylation levels were quantified by bisulfite pyrosequencing. (Ctrl = Folic Acid Control Diet, 7FD = 7x Folic Acid Deficient, 10FS = 10x Folic Acid Supplemented, 20FS = 20x Folic Acid Supplemented).

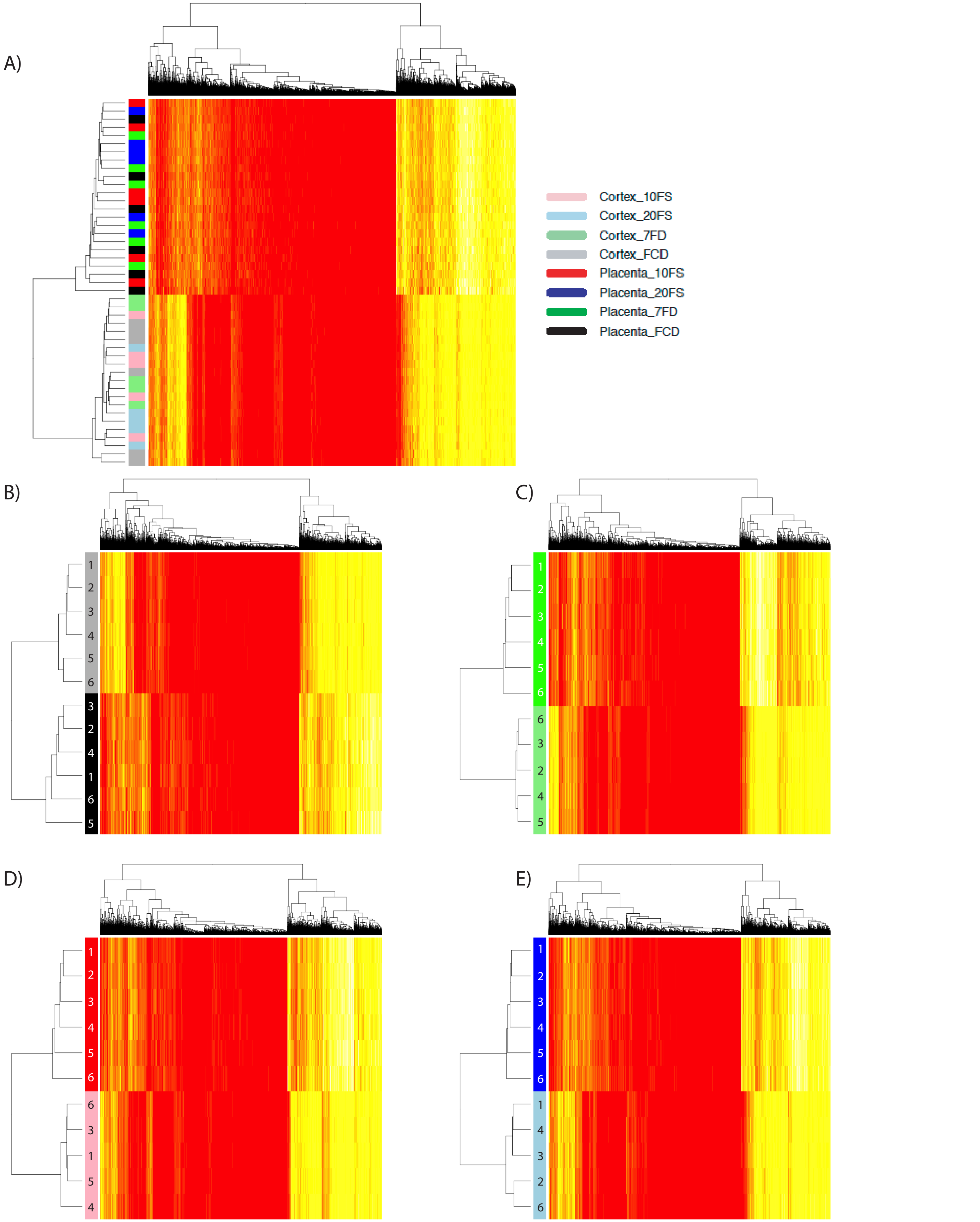

Supplementary Figure S5. Heatmap and hierarchal clustering of commonly sequenced CpG sites at 20x coverage from A) all samples and from cortex and placental samples of B) Ctrl, C) 7FD, D) 10FS and E) 20FS diet groups. Numbers indicate matching cortex and placenta tissues from the same animal. (n = 5-6 males/group; Ctrl = Folic Acid Control Diet, 7FD = 7x Folic Acid Deficient, 10FS = 10x Folic Acid Supplemented, 20FS = 20x Folic Acid Supplemented).

Supplementary Table S1: Pyrosequencing primers for germline differentially methylated regions (DMRs)

Germline DMR (No. of CpGs)	Methylated Allele	Original Sequences of Pyro Primers (Bisufite Converted Sequences)	Reference
H19 (6)	Paternal	Forward: 5'-GGGGGGTAGGATATATGTATTTTT<br>Reverse: 5'-biot-ACCTCATAAAACCCATAACTATAAAATCAT<br>Sequencing: 5'-GTGTGTAAAGATTAGGG	Whidden et al., 2015
SNRPN (5)	Paternal	Forward: 5'-TTGGTAGTTGTTTTTTGGTAGGAT<br>Reverse: 5'-biot-TCCACAAACCCAACTAACCTTC<br>Sequencing: 5'- GTGTAGTTATTGTTTGGGA	Whidden et al., 2015
Peg1 (5)	Maternal	Forward: 5'-GGTTGGGTTTGGATATTGTAAAG<br>Reverse: 5'-biot-TTCCCTAAAATTCTAACAACCTAAACA<br>Sequencing: 5' ATTGTAAAGTTAAAGTTGTAGTAAA	de Waal et al, 2014
Peg3 (6)	Maternal	Forward: 5'-GGTTTTTAAGGGTAATTGATAAGG<br>Reverse: 5'-biot-CCCTATCACCTAAATAACATCCC<br>Sequencing: 5'-AATTGATAAGGTTGTAGATT	de Waal et al, 2015
Kcnq1ot1 (6)	Maternal	Forward: 5'-AGGTTTTGGTAGGTGGTTT<br>Reverse: 5'-biot-CTAACTAAACCAAAATACACCATCATA<br>Sequencing: 5'-GTTAGGAGGAATAGTTGTTTTA	de Waal et al, 2014

H19 Imprinted Maternally Expressed Transcript (Non-Protein Coding)
Snrpn Small Nuclear Ribonucleoprotein Polypeptide N
*Kcnq1ot1*Opposite Strand/Antisense Transcript 1 (Non-Protein Coding)
Peg 1 and 3 Paternally-Expressed Gene 1 and 3

Supplementary Table S2: Summary RRBS performance for individual samples

	Mean	± SEM	Min.	Max.
**Total Reads**	18, 262, 640	374,466	12,777,040	18,971,750
**1× Coverage CpG Count**	1, 629, 169	15, 307	1, 219, 880	1, 971, 469
**CpGs with >=10× Coverage**	64.58%	0.36%	56.76%	69.29%

SEM: Standard Error of Mean; Min.: minimum; Max.: Maximum

Supplementary Table S3: Primers used for the validation of RRBS results.

Pyrosequencing Assay	Target Region	Primers
1	Chr 5:112467185-210	For 5’-biot-GGATTGAGATTGTTGGTTATAAAGG-3’<br>Rev 5’-CCAAAACACTACATTAAAACAAACCCA-3’<br>Seq 5’-AAACCACAAATCATATATAATATC-3’
2	Chr 5: 112467230-250	For 5’- ATGGATTGAGATTGTTGGTTATAAAG -3’<br>Rev 5’-biot-TCCAAAACACTACATTAAAACAAACCC -3’<br>Seq 5’- AGGTGATATTATATATGA -3’
3	Chr x: 95461105-145	For 5’-biot-TGTTGGGGGTATATATGAGGAG -3’<br>Rev 5’- AACTTACCAATACTACTATTCCTCAACTAA -3’<br>Seq 5’- ATCCACAAAATCCTTAAAT -3’
4	Chr 7: 80390945-985	For 5’-biot-GGGGTAGTTTTTTGTAGGAGAT -3’<br>Rev 5’- TCAACAACAACCTCCTACACTA -3’<br>Seq 5’- ACCTCTCACCTACCC -3’

For: Forward PCR primer; **Rev**: Reverse PCR primer; **Seq**: Pyrosequencing primer.

Supplementary Table S4: RRBS comparison of 7FD, 10FS, and 20FS versus Ctrl placenta and cortex

Comparison	Tissue	Number of DMTs		
		Total	Hypermethylation (%)	Hypomethylation (%)
7FD vs Ctrl	Placenta	907	720 (79.4%)	187 (20.6%)
	Cortex	420	325 (77.3%)	95 (22.7%)
10FS vs Ctrl	Placenta	1163	916 (78.7%)	247 (21.3%)
	Cortex	481	290 (60.2%)	191 (39.8%)
20FS vs Ctrl	Placenta	1117	849 (76.0%)	268 (34.0%)
	Cortex	938	307 (32.7%)	631(67.3%)

Supplementary Table S5: Summary Placenta RRBS Data

Values represent average number of tiles of all samples within tissue across all exposures.

		Placenta			
		**All sequenced tiles**	**All DMTs**	**Hypermethylated**	**Hypomethylated**
**Genic Regions**	*Intergenic*	35244 (27%)	225 (47%)	319 (39%)	131 (56%)
	Promoter-TSS	29523 (23%)	22 (3%)	41 (5%)	3 (1%)
	Exon	19871 (15%)	95 (16%)	160 (19%)	29 (12%)
	Intron	36415 (28%)	159 (29%)	253 (39%)	64 (27%)
	Other	9525 (7%)	32 (5%)	45 (6%)	10 (3%)

Supplementary Table S6: Summary Cortex RRBS Data

Values represent average number of tiles of all samples within tissue across all exposures.

		Cortex			
		**All sequenced tiles**	**All DMTs**	**Hypermethylated**	**Hypomethylated**
**Genic Regions**	*Intergenic*	35346 (27%)	131 (44%)	137 (45%)	125 (43%)
	Promoter-TSS	29612 (23%)	10 (3%)	13 (4%)	7 (2%)
	Exon	19945 (15%)	37 (11%)	37 (12%)	36 (11%)
	Intron	36557 (28%)	117 (38%)	108 (35%)	126 (41%)
	Other	9564 (7%)	12 (4%)	14 (4%)	11 (3%)

Supplementary Table S7: Validation of RRBS results by bisulfite pyrosequencing.

Groups compared	Genic Region	Pyrosequencing Assay	CpG Coordinate	RRBS Methylation Change	Pyrosequencing Methylation Change
FCD vs. 7FD	Intron	1	Chr 5: 112467190	↓	↓ [2]
	Intron	1	Chr 5: 112467206	=	= [2]
	Intron	2	Chr 5: 112467237	↓	↓ [2]
	Intron	2	Chr 5: 112467247	↓	↓ [2]
	Intergenic	3	Chr x: 95461111	↓	↓
	Intergenic	3	Chr x: 95461115	↓	↓
	Intergenic	3	Chr x: 95461124	↓	=
	Intergenic	3	Chr x: 95461142	↓	↓
FCD vs. 20FS	Exon	4	Chr 7: 80390950	↑	↑ [1]
	Exon	4	Chr 7: 80390982	↑	↑ [1]

FCD: Folic acid control diet; **7FD**: Seven-fold folic acid deficient diet; **20FS**: Twenty-fold folic acid supplemented diet.
1: Validation performed using 5 samples from the group 20FS. **2**: Validation performed using 5 samples from groups FCD and 7FD.

Supplementary Table S8: Gene lists corresponding to the top 2 most statistically significantly enriched biological pathways among genic DMTs within placenta conserved in all three folic acid exposure groups identified by DAVID Bioinformatic analysis.

Multicellular organism development	Transcription
Alx3	Ccnk
Angpt2	Erbb4
Casz1	Esrrg
Dact2	Fezf2
Dbx1	Gtf2h4
Dll4	Hoxa10
Dlx4	Ncor2
Efnb3	Nfil3
En1	Onecut2
Erbb4	Park2
Esrrg	Pax2
Fezf2	Pou2f2
Hoxa10	Prdm11
Hoxc12	Stat4
Lrp1	Tbr1
Pax2	Tbx1
Six6	Tbx21
Smad5	Vax1
Tbx1	Vopp1
Unc5a	Vsx2
Vax1	Wt1
Vsx2	Ybx1
Wnt2	Zfp423
Wnt7a	Zfp710
Zfa-ps	Zfp784
Zfp423	

Supplementary Table S9: Corresponding genes of DMTs conserved in both placenta and cortex with ≥10% change in methylation. For each gene, the directionality and magnitude of change is demonstrated, along with the diet exposure.

Gene Name	Gene Description	DMT Location	Direction		Diet
			Placenta	Cortex	
Map1lc3b	microtubule-associated protein 1 light chain 3 beta	Intergenic	-21.89	-20.64	7FD
Gm6588	predicted gene 6588	intron	-27.97	-17.61	7FD
Etaa1	Ewing tumor-associated antigen 1	Intergenic	-12.43	-17.16	7FD
Fbxo4	F-box protein 4	Intergenic	-14.12	-16.08	7FD
Sntb1	syntrophin, basic 1	Intergenic	-15.33	-13.58	7FD
2200002D01Rik	RIKEN cDNA 2200002D01 gene	TTS	-21.23	-12.04	7FD
Slc25a13	solute carrier family 25 (mitochondrial carrier, adenine nucleotide translocator), member 13	Intergenic	-10.64	-11.83	7FD
Tas2r139	taste receptor, type 2, member 139	Intergenic	-14.03	-10.81	7FD
1700049E22Rik	RIKEN cDNA 1700049E22 gene	Intergenic	-16.30	-10.61	7FD
Ceacam18	carcinoembryonic antigen-related cell adhesion molecule 18	exon	-15.06	-10.36	7FD
Mtpn	myotrophin	Intergenic	-17.40	-10.11	7FD
Arhgef26	Rho guanine nucleotide exchange factor (GEF) 26	Intergenic	36.55	10.01	7FD
Ccdc166	coiled-coil domain containing 166	exon	15.21	10.07	7FD
Ybx1	Y box protein 1	exon	20.17	10.17	7FD
Fam58b	family with sequence similarity 58, member B	exon	12.31	10.20	7FD
Ckb	creatine kinase, brain	intron	15.42	10.70	7FD
Mir6943	microRNA mir-6943	intron	13.61	10.73	7FD
Slc38a1	solute carrier family 38, member 1	Intergenic	10.57	10.78	7FD
Olfr136	olfactory receptor 136	Intergenic	21.38	10.92	7FD
Gnas	GNAS (guanine nucleotide binding protein, alpha stimulating) complex locus	exon	25.01	11.17	7FD
Esrrg	estrogen-related receptor gamma	intron	10.24	11.18	7FD
1700030C10Rik	RIKEN cDNA 1700030C10 gene	Intergenic	12.44	11.36	7FD
Prok2	prokineticin 2	Intergenic	13.24	11.42	7FD
Smad1	SMAD family member 1	intron	15.48	11.63	7FD
Alkbh5	alkB, alkylation repair homolog 5 (E. coli)	Intergenic	15.27	11.64	7FD
Arhgef28	Rho guanine nucleotide exchange factor (GEF) 28	intron	11.92	12.01	7FD
Cdh15	cadherin 15	intron	14.54	12.48	7FD
Sall1	sal-like 1 (Drosophila)	Intergenic	10.26	12.62	7FD
Aif1l	allograft inflammatory factor 1-like	Intergenic	12.82	12.65	7FD
Fam19a5	family with sequence similarity 19, member A5	Intergenic	-10.81	12.67	7FD
Cd209c	CD209c antigen	Intergenic	17.80	12.80	7FD
Tmem63a	transmembrane protein 63a	3' UTR	21.12	13.20	7FD
Anks6	ankyrin repeat and sterile alpha motif domain containing 6	exon	12.37	13.38	7FD
Pex14	peroxisomal biogenesis factor 14	3' UTR	18.17	14.66	7FD
Mir378c	microRNA mir-378c	intron	12.55	14.71	7FD
Ckb	creatine kinase, brain	exon	25.05	14.76	7FD
Pcdh19	protocadherin 19	Intergenic	10.10	15.20	7FD

Table S9 continued

Scgb2b26	secretoglobin, family 2B, member 26	Intergenic	10.73	15.40	7FD
Fam58b	family with sequence similarity 58, member B	exon	23.31	15.52	7FD
Olfm1	olfactomedin 1	Intergenic	10.92	15.76	7FD
Stxbp6	syntaxin binding protein 6 (amisyn)	Intergenic	17.69	16.11	7FD
Mir7214	microRNA mir-7214	Intergenic	16.39	16.43	7FD
Ybx1	Y box protein 1	exon	17.68	17.71	7FD
Ccnk	cyclin K	intron	15.89	21.73	7FD
Mir153	microRNA 153	intron	14.09	23.15	7FD
Eltd1	EGF, latrophilin seven transmembrane domain containing 1	Intergenic	13.46	26.73	7FD

Map1lc3b	microtubule-associated protein 1 light chain 3 beta	Intergenic	-22.78	-18.38	10FS
Sntb1	syntrophin, basic 1	Intergenic	-11.09	-17.82	10FS
Six3os1	SIX homeobox 3, opposite strand 1	Intergenic	12.71	-17.43	10FS
2610316D01Rik	RIKEN cDNA 2610316D01 gene	Intergenic	-19.01	-16.24	10FS
Mif	macrophage migration inhibitory factor	intron	20.52	-15.79	10FS
Etaa1	Ewing tumor-associated antigen 1	Intergenic	-15.82	-12.35	10FS
Sh3pxd2a	SH3 and PX domains 2A	intron	-11.80	-11.46	10FS
Krtcap3	keratinocyte associated protein 3	intron	-15.99	-11.32	10FS
Gpr110	G protein-coupled receptor 110	Intergenic	-13.73	-11.28	10FS
Rps6kc1	ribosomal protein S6 kinase polypeptide 1	Intergenic	-17.30	-10.27	10FS
2200002D01Rik	RIKEN cDNA 2200002D01 gene	TTS	-15.91	-10.17	10FS
Psg16	pregnancy specific glycoprotein 16	intron	14.10	-10.12	10FS
Gnas	GNAS (guanine nucleotide binding protein, alpha stimulating) complex locus	exon	11.13	-10.02	10FS
Ino80d	INO80 complex subunit D	Intergenic	13.27	10.20	10FS
Ppp1r2-ps3	protein phosphatase 1, regulatory (inhibitor) subunit 2, pseudogene 3	promoter-TSS	33.06	10.33	10FS
Rpp21	ribonuclease P 21 subunit	Intergenic	14.19	10.43	10FS
H2-DMa	histocompatibility 2, class II, locus DMa	intron	12.24	10.58	10FS
LOC100503676	uncharacterized LOC100503676	intron	13.75	10.66	10FS
Alkbh5	alkB, alkylation repair homolog 5 (E. coli)	Intergenic	17.31	10.78	10FS
1700034G24Rik	RIKEN cDNA 1700034G24 gene	Intergenic	16.47	10.97	10FS
Anks6	ankyrin repeat and sterile alpha motif domain containing 6	exon	12.29	11.11	10FS
Olfr136	olfactory receptor 136	Intergenic	18.56	11.13	10FS
Farp1	FERM, RhoGEF (Arhgef) and pleckstrin domain protein 1 (chondrocyte-derived)	Intergenic	10.87	11.23	10FS
Hrh2	histamine receptor H2	Intergenic	13.71	11.36	10FS
Fam58b	family with sequence similarity 58, member B	exon	16.40	11.37	10FS
Rcan2	regulator of calcineurin 2	intron	16.56	11.46	10FS
Neurl1a	neuralized homolog 1A (Drosophila)	exon	11.36	11.61	10FS
Nenf	neuron derived neurotrophic factor	Intergenic	-11.22	11.85	10FS
1700030C10Rik	RIKEN cDNA 1700030C10 gene	Intergenic	13.49	11.91	10FS
Mup5	major urinary protein 5	Intergenic	18.71	12.22	10FS
Mir7032	microRNA mir-7032	intron	11.61	12.68	10FS
1700010K23Rik	RIKEN cDNA 1700010K23 gene	intron	26.82	12.97	10FS
Prok2	prokineticin 2	Intergenic	16.24	13.08	10FS
Stxbp6	syntaxin binding protein 6 (amisyn)	Intergenic	15.56	13.11	10FS

Table S9 continued

Kit	kit oncogene	intron	12.23	13.45	10FS
Ereg	epiregulin	promoter-TSS	11.23	14.18	10FS
Ckb	creatine kinase, brain	exon	24.94	14.31	10FS
Aif1l	allograft inflammatory factor 1-like	Intergenic	14.86	14.66	10FS
Mir7214	microRNA mir-7214	Intergenic	15.72	15.42	10FS
Arhgef26	Rho guanine nucleotide exchange factor (GEF) 26	Intergenic	28.44	15.50	10FS
Scgb2b26	secretoglobin, family 2B, member 26	Intergenic	15.82	15.62	10FS
Gdnf	glial cell line derived neurotrophic factor	intron	10.85	15.95	10FS
Ccnk	cyclin K	intron	15.99	15.96	10FS
Pisd-ps3	phosphatidylserine decarboxylase, pseudogene 3	promoter-TSS	10.04	16.50	10FS
Sipa1	signal-induced proliferation associated gene 1	exon	17.11	17.52	10FS
Eltd1	EGF, latrophilin seven transmembrane domain containing 1	Intergenic	12.96	17.72	10FS
Ube2v1	ubiquitin-conjugating enzyme E2 variant 1	Intergenic	13.55	18.10	10FS
Rpp21	ribonuclease P 21 subunit	Intergenic	-12.37	18.21	10FS
Arhgef26	Rho guanine nucleotide exchange factor (GEF) 26	Intergenic	17.86	18.46	10FS
Mark4	MAP/microtubule affinity-regulating kinase 4	Intergenic	17.13	19.03	10FS
Irgq	immunity-related GTPase family, Q	exon	12.04	19.19	10FS
Esp38	exocrine gland secreted peptide 38	Intergenic	14.41	21.06	10FS
Mir8110	microRNA mir-8110	Intergenic	18.39	22.40	10FS
Rab11fip3	RAB11 family interacting protein 3 (class II)	intron	17.99	28.26	10FS
Efhc2	EF-hand domain (C-terminal) containing 2	intron	13.31	29.55	10FS

Mif	macrophage migration inhibitory factor	intron	28.35	-27.45	20FS
Map1lc3b	microtubule-associated protein 1 light chain 3 beta	Intergenic	-25.52	-22.70	20FS
Six3os1	SIX homeobox 3, opposite strand 1	Intergenic	12.55	-21.17	20FS
Zc3h10	zinc finger CCCH type containing 10	promoter-TSS	-13.71	-17.57	20FS
2610316D01Rik	RIKEN cDNA 2610316D01 gene	Intergenic	-20.18	-17.44	20FS
Gm20319	predicted gene, 20319	exon	-11.45	-14.58	20FS
1700086O06Rik	RIKEN cDNA 1700086O06 gene	Intergenic	10.08	-13.84	20FS
Gpr110	G protein-coupled receptor 110	Intergenic	-15.25	-13.21	20FS
Krtcap3	keratinocyte associated protein 3	intron	-15.29	-13.13	20FS
2200002D01Rik	RIKEN cDNA 2200002D01 gene	TTS	-19.74	-12.39	20FS
Clmn	calmin	Intergenic	11.22	-12.20	20FS
Htr1a	5-hydroxytryptamine (serotonin) receptor 1A	Intergenic	14.23	-11.96	20FS
Mtpn	myotrophin	Intergenic	-15.10	-11.61	20FS
Sntb1	syntrophin, basic 1	Intergenic	-12.66	-11.59	20FS
Tas1r2	taste receptor, type 1, member 2	Intergenic	13.82	-11.52	20FS
Ear2	eosinophil-associated, ribonuclease A family, member 2	Intergenic	-11.04	-11.36	20FS
Eva1b	eva-1 homolog B (C. elegans)	TTS	12.76	-11.16	20FS
Myog	myogenin	3' UTR	17.56	-10.98	20FS
Slc25a13	solute carrier family 25 (mitochondrial carrier, adenine nucleotide translocator), member 13	Intergenic	-10.61	-10.62	20FS
Slc28a3	solute carrier family 28 (sodium-coupled nucleoside transporter), member 3	intron	12.99	-10.44	20FS

Table S9 continued

Gdf2	growth differentiation factor 2	exon	11.20	-10.38	20FS
Gm10377	predicted gene 10377	Intergenic	-13.93	-10.29	20FS
Chsy3	chondroitin sulfate synthase 3	intron	-14.35	-10.28	20FS
Gm13152	predicted gene 13152	intron	11.25	10.05	20FS
Olfr136	olfactory receptor 136	Intergenic	22.33	10.07	20FS
Tbce	tubulin-specific chaperone E	intron	25.53	10.21	20FS
Pim1	proviral integration site 1	Intergenic	14.48	10.31	20FS
Arhgef26	Rho guanine nucleotide exchange factor (GEF) 26	Intergenic	20.57	10.61	20FS
Rcan2	regulator of calcineurin 2	intron	11.33	10.77	20FS
Anks6	ankyrin repeat and sterile alpha motif domain containing 6	exon	17.85	11.03	20FS
Car10	carbonic anhydrase 10	Intergenic	17.38	11.12	20FS
Ncor2	nuclear receptor co-repressor 2	Intergenic	18.34	11.15	20FS
Ephb3	Eph receptor B3	promoter-TSS	13.32	11.16	20FS
Fbxo16	F-box protein 16	intron	-12.33	11.31	20FS
Cyp51	cytochrome P450, family 51	Intergenic	10.42	11.46	20FS
Piezo1	piezo-type mechanosensitive ion channel component 1	intron	11.88	11.53	20FS
Stc2	stanniocalcin 2	Intergenic	-23.84	11.85	20FS
Arhgef26	Rho guanine nucleotide exchange factor (GEF) 26	Intergenic	39.30	12.01	20FS
Ckb	creatine kinase, brain	exon	32.09	12.02	20FS
Stxbp6	syntaxin binding protein 6 (amisyn)	Intergenic	23.25	12.72	20FS
Ybx1	Y box protein 1	exon	16.99	12.76	20FS
Pex2	peroxisomal biogenesis factor 2	Intergenic	-14.17	12.85	20FS
Erdr1	erythroid differentiation regulator 1	Intergenic	-10.82	13.10	20FS
Ppp1r2-ps3	protein phosphatase 1, regulatory (inhibitor) subunit 2, pseudogene 3	promoter-TSS	36.40	13.22	20FS
LOC100503676	uncharacterized LOC100503676	intron	10.79	13.36	20FS
Kcns1	K+ voltage-gated channel, subfamily S, 1	5' UTR	12.58	13.41	20FS
Spred2	sprouty-related, EVH1 domain containing 2	intron	12.93	13.64	20FS
Eltd1	EGF, latrophilin seven transmembrane domain containing 1	Intergenic	18.02	13.70	20FS
Scrg1	scrapie responsive gene 1	intron	16.52	13.73	20FS
Aif1l	allograft inflammatory factor 1-like	Intergenic	12.08	14.06	20FS
Mark4	MAP/microtubule affinity-regulating kinase 4	Intergenic	25.01	14.15	20FS
Pax7	paired box 7	exon	10.88	14.28	20FS
A930016O22Rik	RIKEN cDNA A930016O22 gene	intron	14.62	14.36	20FS
Proser2	proline and serine rich 2	exon	-10.04	14.39	20FS
Scgb2b26	secretoglobin, family 2B, member 26	Intergenic	13.55	14.55	20FS
Aldh2	aldehyde dehydrogenase 2, mitochondrial	intron	12.94	14.66	20FS
Ptprtos	protein tyrosine phosphatase, receptor type T, opposite strand	intron	-18.15	15.28	20FS
Hmgn2	high mobility group nucleosomal binding domain 2	intron	12.43	15.41	20FS
Hao1	hydroxyacid oxidase 1, liver	intron	-16.85	16.22	20FS
Irgq	immunity-related GTPase family, Q	exon	14.01	16.48	20FS
4921524L21Rik	RIKEN cDNA 4921524L21 gene	Intergenic	10.97	16.80	20FS
Psg16	pregnancy specific glycoprotein 16	intron	16.75	17.59	20FS

Table S9 continued

Mir7214	microRNA mir-7214	Intergenic	18.32	18.01	20FS
Esp38	exocrine gland secreted peptide 38	Intergenic	-11.34	18.29	20FS
Sipa1	signal-induced proliferation associated gene 1	exon	20.13	18.91	20FS
Tshz3	teashirt zinc finger family member 3	Intergenic	29.74	19.27	20FS
Rab11fip3	RAB11 family interacting protein 3 (class II)	intron	17.66	21.82	20FS
Ap5z1	adaptor-related protein complex 5, zeta 1 subunit	intron	27.76	21.94	20FS
Rpp21	ribonuclease P 21 subunit	Intergenic	-22.79	22.09	20FS
Mir153	microRNA 153	intron	10.95	34.38	20FS
Rasa1	RAS p21 protein activator 1	Intergenic	-16.35	35.42	20FS

Supplementary Table S10: Placenta (A) and cortex (B) RRBS DMTs localized to regions in previously published sequencing data with low methylation in sperm (≤10%) but moderate to high methylation in germinal vesicle oocytes (≥25%) and inner cell mass (≥25%)

(A)

	Placenta					
	7FD		10FS		20FS	
**DMT Direction**	**Hyper**	**Hypo**	**Hyper**	**Hypo**	**Hyper**	**Hypo**
**Total**	44	6	63	4	44	11
**% Genic (# DMTs)**	77.3 (34)	83.3 (5)	82.5 (52)	100 (4)	86.4 (38)	90 (10)

(B)

	Cortex					
	7FD		10FS		20FS	
**DMT Direction**	**Hyper**	**Hypo**	**Hyper**	**Hypo**	**Hyper**	**Hypo**
**Total**	16	3	17	5	13	24
**% Genic (# DMTs)**	81.3 (13)	66.7 (2)	58.8 (10)	80 (4)	76.9 (10)	87.5 (21)

Supplementary Table S11

List of DMTs with location, closest associated Ensembl gene ID, degree of alteration in DNA methylation and the level of methylation of the loci taken from the published GVO and ICMm data.

Tissue	Diet	Location (chr:start-stop)	Peak_Score	Annotation	Gene_ID	GVO	ICMm
Cortex	7FD	chr1:171287801-171287900	23.1	TTS	Usp21	69.5	33.5
		chr1:171287901-171288000	24.4	promoter-TSS	Usp21	69.5	33.5
		chr19:30539701-30539800	15.2	promoter-TSS	Ppp1r2-ps3	92.5	28.2
		chr15:72809801-72809900	10.8	non-coding	Peg13	96.8	86.0
		chr15:72809701-72809800	10.6	non-coding	Peg13	96.8	86.0
		chrX:169983701-169983800	11.0	intron	G530011O06Rik	57.0	34.9
		chr5:125612501-125612600	23.9	intron	Tmem132b	95.3	59.2
		chr18:75258801-75258900	14.2	intron	2010010A06Rik	96.5	56.9
		chr7:82771901-82772000	17.0	intron	4933406J10Rik	95.3	48.0
		chr16:89954801-89954900	18.3	intron	Tiam1	95.2	45.2
		chr3:62159001-62159100	10.0	Intergenic	Arhgef26	84.2	86.2
		chr15:84417201-84417300	12.0	exon	1810041L15Rik	90.9	57.6
		chr13:95763201-95763300	23.0	exon	F2rl2	97.6	56.8
		chr8:108956801-108956900	10.0	exon	Mir3108	96.5	49.3
		chr4:148953801-148953900	14.7	3' UTR	Pex14	74.0	29.7
		chr5:24482501-24482600	11.2	3' UTR	Agap3	90.4	41.1
		chr17:47867001-47867100	-12.1	TTS	Mdfi	79.5	37.5
		chr16:11143901-11144000	-12.0	intron	Txndc11	36.7	32.1
		chr12:24493501-24493600	-15.7	Intergenic	Taf1b	86.1	36.5
	10FS	chr7:4866401-4866500	20.4	promoter-TSS	Isoc2b	85.1	43.4
		chr19:30539701-30539800	10.3	promoter-TSS	Ppp1r2-ps3	92.5	28.2
		chr16:11144101-11144200	11.7	intron	Txndc11	36.7	32.1
		chr7:44630401-44630500	10.7	intron	Myh14	45.2	32.8
		chr10:13091001-13091100	18.5	intron	Plagl1	96.9	64.3
		chr15:80367001-80367100	10.9	intron	Cacna1i	96.7	62.4
		chr7:82771901-82772000	14.4	intron	4933406J10Rik	95.3	48.0
		chr3:62159401-62159500	18.5	Intergenic	Arhgef26	84.2	86.2
		chr3:62159001-62159100	15.5	Intergenic	Arhgef26	84.2	86.2
		chr11:116498701-116498800	13.1	Intergenic	Prpsap1	47.4	41.7
		chrX:169994101-169994200	15.6	Intergenic	G530011O06Rik	57.0	34.9
		chr14:65111001-65111100	27.9	Intergenic	Extl3	96.0	71.2
		chr8:108241501-108241600	14.4	Intergenic	3010033K07Rik	95.2	47.5
		chr8:108241401-108241500	11.4	Intergenic	3010033K07Rik	95.2	47.5
		chr19:5660901-5661000	17.5	exon	Sipa1	96.8	65.6
		chr15:84417201-84417300	14.8	exon	1810041L15Rik	90.9	57.6
		chr1:15892401-15892500	10.1	exon	Sbspon	96.2	49.3
		chr4:154087101-154087200	-11.6	intron	Trp73	97.3817838	52.5
		chr11:116991601-116991700	-19.2	Intergenic	Mgat5b	79.1751366	36.0
		chr11:101165501-101165600	-11.4	exon	Plekhh3	96.6450234	58.0
		chr11:116009901-116010000	-12.0	exon	Galk1	87.8266254	44.5
		chr16:43974001-43974100	-10.8	exon	Zdhhc23	97.1549415	51.4
	20FS	chr19:30539701-30539800	13.2	promoter-TSS	Ppp1r2-ps3	92.5	28.2
		chr16:11144101-11144200	15.7	intron	Txndc11	36.7	32.1
		chr7:44630401-44630500	13.1	intron	Myh14	45.2	32.8
		chrX:169983701-169983800	39.0	intron	G530011O06Rik	57.0	34.9
		chr15:80367001-80367100	10.1	intron	Cacna1i	96.7	62.4
		chr7:82771901-82772000	26.4	intron	4933406J10Rik	95.3	48.0
		chr16:89954801-89954900	16.3	intron	Tiam1	95.2	45.2
		chr3:62159001-62159100	12.0	Intergenic	Arhgef26	84.2	86.2
		chr3:62159401-62159500	10.6	Intergenic	Arhgef26	84.2	86.2
		chr8:108241501-108241600	11.6	Intergenic	3010033K07Rik	95.2	47.5
		chr19:5660901-5661000	18.9	exon	Sipa1	96.8	65.6
		chr15:84417201-84417300	17.9	exon	1810041L15Rik	90.9	57.6
		chr13:95763201-95763300	30.6	exon	F2rl2	97.6	56.8
		chr11:116009901-116010000	-14.5	exon	Galk1	87.8	44.5

Table S11 continued

		chr8:70392901-70393000	-10.3	exon	Comp	96.1	48.4
		chr5:115231401-115231500	-11.0	Intergenic	Pop5	95.4	53.6
		chr5:115231301-115231400	-12.7	Intergenic	Pop5	95.4	53.6
		chr5:113193901-113194000	-13.2	Intergenic	2900026A02Rik	97.0	45.5
		chr2:31495801-31495900	-11.6	intron	Gm5424	95.4	63.2
		chr5:117622301-117622400	-13.0	intron	Ksr2	96.1	62.9
		chr5:125612401-125612500	-15.0	intron	Tmem132b	95.3	59.2
		chr11:108940301-108940400	-15.8	intron	Axin2	96.7	59.7
		chr12:108259801-108259900	-12.8	intron	Ccdc85c	96.5	58.2
		chr1:36721601-36721700	-10.7	intron	4933424G06Rik	92.6	48.2
		chr8:84731601-84731700	-11.1	intron	Lyl1	87.7	42.8
		chr8:84722001-84722100	-13.8	intron	Lyl1	87.7	42.8
		chr8:108920001-108920100	-11.9	intron	Mir3108	96.5	49.3
		chr1:133068901-133069000	-10.6	intron	Pik3c2b	97.4	42.8
		chr5:113126501-113126600	-12.7	intron	2900026A02Rik	96.9	42.2
		chr5:113126601-113126700	-15.1	intron	2900026A02Rik	96.9	42.2
		chr15:55121401-55121500	-12.2	intron	Gm9920	92.6	37.5
		chr10:127653401-127653500	-12.8	intron	Stat6	86.0	30.3
		chr17:28023101-28023200	-17.0	intron	Tcp11	97.3	33.1
		chr17:63937801-63937900	-16.8	promoter-TSS	Fert2	96.3	33.4
		chr4:155740001-155740100	-11.3	TTS	Tmem240	72.8	47.6
		chr8:70331301-70331400	-11.8	TTS	Gdf1	89.3	53.1
		chr17:47867001-47867100	-16.2	TTS	Mdfi	79.5	37.5
**Placenta**	**7FD**	chr4:148952901-148953000	21.0	3' UTR	Pex14	74.0	29.7
		chr4:148953801-148953900	18.2	3' UTR	Pex14	74.0	29.7
		chr11:69560001-69560100	13.5	5' UTR	Efnb3	95.0	36.2
		chr15:81235701-81235800	16.3	5' UTR	Mchr1	96.8	44.2
		chr9:31349201-31349300	14.4	exon	Prdm10	96.7	31.4
		chr5:137315301-137315400	12.6	exon	Trip6	93.2	35.2
		chr10:7867601-7867700	15.0	exon	Mir5104	49.6	37.4
		chr10:7867501-7867600	12.4	exon	Mir5104	49.6	37.4
		chr8:121753501-121753600	11.0	exon	Jph3	89.1	39.1
		chr8:121753201-121753300	10.7	exon	Jph3	89.1	39.1
		chr11:101265401-101265500	14.1	exon	Wnk4	89.6	41.2
		chr5:110342201-110342300	10.5	exon	P2rx2	90.8	46.3
		chr11:116145701-116145800	14.6	exon	Mrpl38	97.2	46.3
		chr1:134987601-134987700	10.6	exon	Ube2t	95.6	53.4
		chr5:24414001-24414100	12.1	exon	Asic3	93.8	54.8
		chr10:127049701-127049800	12.0	exon	Cyp27b1	95.0	56.4
		chr4:130072801-130072900	10.0	exon	Col16a1	91.2	56.6
		chr2:162948501-162948600	10.7	exon	L3mbtl1	96.8	64.7
		chr19:5660901-5661000	17.4	exon	Sipa1	96.8	65.6
		chr19:5660801-5660900	11.5	exon	Sipa1	96.8	65.6
		chr5:114923401-114923500	10.8	exon	Oasl1	96.7	73.8
		chr6:54021401-54021500	17.7	Intergenic	Chn2	83.2	35.3
		chr2:44556901-44557000	20.2	Intergenic	Gtdc1	79.1	35.7
		chr2:44557001-44557100	11.6	Intergenic	Gtdc1	79.1	35.7
		chr11:116994801-116994900	13.4	Intergenic	Mgat5b	79.2	36.0
		chr13:65258901-65259000	10.7	Intergenic	Zfp369	51.0	37.8
		chr11:19475601-19475700	14.8	Intergenic	4933406G16Rik	93.6	51.3
		chr3:62159001-62159100	36.5	Intergenic	Arhgef26	84.2	86.2
		chr3:62159401-62159500	19.3	Intergenic	Arhgef26	84.2	86.2
		chr10:127620101-127620200	11.2	intron	Lrp1	75.6	32.9
		chr17:28013801-28013900	16.1	intron	Tcp11	97.3	33.1
		chr7:16858501-16858600	13.3	intron	Prkd2	96.2	36.3
		chr10:11449801-11449900	10.0	intron	Epm2a	94.5	39.6
		chr8:87938601-87938700	14.7	intron	Zfp423	93.5	41.2
		chr11:69399801-69399900	12.6	intron	Tmem88	96.2	44.8
		chr5:35763001-35763100	11.7	intron	Ablim2	88.8	46.1
		chr1:52009101-52009200	10.5	intron	Stat4	95.1	52.8
		chr4:148108001-148108100	13.4	intron	Agtrap	91.0	53.8
		chr5:111273701-111273800	11.8	intron	Pitpnb	95.8	62.4
		chr2:168553601-168553700	15.0	intron	Nfatc2	97.3	64.1
		chr3:93214101-93214200	10.4	intron	Flg2	57.8	71.6
		chr6:148355301-148355400	11.8	non-coding	Rps4l	94.6	32.8
		chr6:148355401-148355500	10.6	non-coding	Rps4l	94.6	32.8
		chr5:114806701-114806800	15.5	TTS	1500011B03Rik	96.6	57.4

Table S11 continued

		chr11:96712501-96712600	-13.9	intron	Snx11	95.6	65.6
		chr1:134997401-134997500	-10.9	intron	Ube2t	95.6	53.4
		chr2:20235701-20235800	-13.8	Intergenic	Etl4	30.0	33.7
		chr4:154341601-154341700	-14.5	exon	Arhgef16	94.2	56.3
		chr17:47877101-47877200	-10.1	exon	Mdfi	95.4	47.4
		chr1:34807101-34807200	-11.6	exon	Arhgef4	97.3	45.2
	10FS	chr4:148952901-148953000	20.7	3' UTR	Pex14	74.0	29.7
		chr4:148953801-148953900	12.3	3' UTR	Pex14	74.0	29.7
		chr4:148952601-148952700	11.8	3' UTR	Pex14	74.0	29.7
		chr4:133527901-133528000	10.9	5' UTR	1810019J16Rik	81.3	34.1
		chr11:69560001-69560100	21.8	5' UTR	Efnb3	95.0	36.2
		chr15:81235701-81235800	10.1	5' UTR	Mchr1	96.8	44.2
		chr9:31349201-31349300	14.8	exon	Prdm10	96.7	31.4
		chr4:148952201-148952300	15.4	exon	Pex14	74.0	29.7
		chr4:148952501-148952600	11.8	exon	Pex14	74.0	29.7
		chr4:148952101-148952200	10.3	exon	Pex14	74.0	29.7
		chr19:5660901-5661000	17.1	exon	Sipa1	96.8	65.6
		chr10:127049701-127049800	12.1	exon	Cyp27b1	95.0	56.4
		chr13:21363801-21363900	11.8	exon	Zscan12	95.0	35.2
		chr8:121753101-121753200	11.5	exon	Jph3	89.1	39.1
		chr11:117873201-117873300	19.6	exon	Tha1	88.7	45.8
		chr7:44864901-44865000	13.5	exon	Ptov1	95.9	60.4
		chr11:116145901-116146000	15.4	exon	Mrpl38	97.2	46.3
		chr10:18526901-18527000	10.4	exon	Hebp2	95.7	54.7
		chr11:101265301-101265400	10.9	exon	Wnk4	89.6	41.2
		chr7:30860701-30860800	10.2	exon	Ffar1	92.6	30.5
		chr4:134569001-134569100	14.9	exon	Mir6403	96.7	57.2
		chr6:54021401-54021500	14.1	Intergenic	Chn2	83.2	35.3
		chr2:44556901-44557000	18.6	Intergenic	Gtdc1	79.1	35.7
		chr2:44557001-44557100	17.8	Intergenic	Gtdc1	79.1	35.7
		chr5:111571001-111571100	11.0	Intergenic	C130026L21Rik	90.6	49.4
		chr1:86313701-86313800	10.8	Intergenic	B3gnt7	59.0	49.6
		chr11:19475601-19475700	10.7	Intergenic	4933406G16Rik	93.6	51.3
		chr12:12810101-12810200	11.4	Intergenic	Mycn	96.2	55.8
		chr1:6441401-6441500	12.5	Intergenic	St18	94.9	63.2
		chr3:62159001-62159100	28.4	Intergenic	Arhgef26	84.2	86.2
		chr3:62159401-62159500	17.9	Intergenic	Arhgef26	84.2	86.2
		chr2:168553601-168553700	13.6	intron	Nfatc2	97.3	64.1
		chr12:108887301-108887400	11.3	intron	Wars	96.1	58.0
		chr5:111273701-111273800	13.6	intron	Pitpnb	95.8	62.4
		chr4:136426901-136427000	12.0	intron	Htr1d	96.7	60.2
		chr5:74230201-74230300	17.4	intron	Rasl11b	95.7	53.5
		chr8:108901501-108901600	13.9	intron	Mir3108	96.2	46.7
		chr10:4709901-4710000	10.8	intron	Esr1	96.2	56.2
		chr10:127620101-127620200	12.3	intron	Lrp1	75.6	32.9
		chr5:125058001-125058100	10.7	intron	Fam101a	96.7	54.7
		chr1:52009101-52009200	10.2	intron	Stat4	95.1	52.8
		chr11:98721401-98721500	16.2	intron	Med24	91.1	41.5
		chr3:67462601-67462700	11.2	intron	Lxn	96.4	48.9
		chr11:117873101-117873200	25.2	intron	Tha1	88.7	45.8
		chr8:87938601-87938700	12.7	intron	Zfp423	93.5	41.2
		chr8:87938501-87938600	12.1	intron	Zfp423	93.5	41.2
		chr10:75667701-75667800	12.8	intron	Susd2	95.2	28.7
		chr11:119257201-119257300	12.9	intron	Gaa	78.5	43.9
		chr11:119257301-119257400	12.4	intron	Gaa	78.5	43.9
		chr5:124011001-124011100	10.6	intron	Mir7032	96.2	67.8
		chr7:16858501-16858600	18.9	intron	Prkd2	96.2	36.3
		chr10:127527501-127527600	12.1	intron	Shmt2	60.5	26.6
		chr5:119687101-119687200	12.3	intron	Tbx3os2	86.2	38.9
		chr5:119687301-119687400	11.9	intron	Tbx3os2	86.2	38.9
		chr5:119626801-119626900	11.9	intron	Gm16063	96.0	37.3
		chr11:85800001-85800100	17.9	intron	Bcas3os2	93.7	51.1
		chr6:148355201-148355300	12.5	non-coding	Rps4l	94.6	32.8
		chr10:127642901-127643000	10.2	promoter-TSS	Stat6	86.0	30.3
		chr1:171287901-171288000	15.2	promoter-TSS	Usp21	69.5	33.5
		chr19:30539601-30539700	27.7	promoter-TSS	Ppp1r2-ps3	92.5	28.2
		chr19:30539701-30539800	33.1	promoter-TSS	Ppp1r2-ps3	92.5	28.2

Table S11 continued

		chr1:171287801-171287900	19.0	TTS	Usp21	69.5	33.5
		chr5:114806601-114806700	12.9	TTS	1500011B03Rik	96.6	57.4
		chr11:96712501-96712600	-11.4	intron	Snx11	95.6203577	65.6
		chr4:136222801-136222900	-10.3	intron	Asap3	69.2940039	25.6
		chr17:3350001-3350100	-11.4	intron	Tiam2	96.15595	51.7
		chr13:45567501-45567600	-11.0	exon	Gmpr	97.0248411	49.0
	**20FS**	chr4:148953801-148953900	23.7	3' UTR	Pex14	74.0	29.7
		chr4:148952901-148953000	13.6	3' UTR	Pex14	74.0	29.7
		chr1:134987601-134987700	10.9	exon	Ube2t	95.6	53.4
		chr1:134987701-134987800	17.3	exon	Ube2t	95.6	53.4
		chr2:162948501-162948600	11.7	exon	L3mbtl1	96.8	64.7
		chr4:148952401-148952500	10.7	exon	Pex14	74.0	29.7
		chr4:148952301-148952400	10.2	exon	Pex14	74.0	29.7
		chr4:148952501-148952600	10.0	exon	Pex14	74.0	29.7
		chr19:5660901-5661000	20.1	exon	Sipa1	96.8	65.6
		chr19:5660801-5660900	10.2	exon	Sipa1	96.8	65.6
		chr7:44629901-44630000	16.3	exon	Myh14	45.2	32.8
		chr10:127049701-127049800	14.0	exon	Cyp27b1	95.0	56.4
		chr8:121753201-121753300	11.7	exon	Jph3	89.1	39.1
		chr8:121753501-121753600	11.0	exon	Jph3	89.1	39.1
		chr2:145860701-145860800	10.2	exon	Naa20	89.6	40.4
		chr10:7867601-7867700	13.2	exon	Mir5104	49.6	37.4
		chr11:101265401-101265500	10.6	exon	Wnk4	89.6	41.2
		chr11:101265301-101265400	10.0	exon	Wnk4	89.6	41.2
		chr4:134569001-134569100	18.9	exon	Mir6403	96.7	57.2
		chr3:62159001-62159100	39.3	Intergenic	Arhgef26	84.2	86.2
		chr3:62159401-62159500	20.6	Intergenic	Arhgef26	84.2	86.2
		chr18:83062201-83062300	11.0	Intergenic	4930592I03Rik	84.2	63.2
		chr2:44556901-44557000	14.1	Intergenic	Gtdc1	79.1	35.7
		chr6:54021401-54021500	20.5	Intergenic	Chn2	83.2	35.3
		chr11:58960301-58960400	11.4	Intergenic	Trim17	56.0	33.3
		chr11:69399801-69399900	10.8	intron	Tmem88	96.2	44.8
		chr17:28013801-28013900	10.3	intron	Tcp11	97.3	33.1
		chr10:127620101-127620200	12.5	intron	Lrp1	75.6	32.9
		chr10:127620201-127620300	12.1	intron	Lrp1	75.6	32.9
		chr10:11449801-11449900	13.1	intron	Epm2a	94.5	39.6
		chr1:52009101-52009200	10.8	intron	Stat4	95.1	52.8
		chr5:24332101-24332200	13.4	intron	Kcnh2	82.8	44.1
		chr4:148108001-148108100	11.0	intron	Agtrap	91.0	53.8
		chr8:87938601-87938700	19.8	intron	Zfp423	93.5	41.2
		chr8:87938501-87938600	14.4	intron	Zfp423	93.5	41.2
		chr11:88402501-88402600	10.9	intron	Mir378b	79.6	37.2
		chr10:75667701-75667800	18.1	intron	Susd2	95.2	28.7
		chr6:116647901-116648000	10.7	intron	Mir7043	64.9	47.8
		chr7:16858501-16858600	18.8	intron	Prkd2	96.2	36.3
		chr7:16853601-16853700	10.8	intron	Prkd2	96.2	36.3
		chr17:63937901-63938000	21.0	promoter-TSS	Fert2	96.3	33.4
		chr7:60005101-60005200	10.3	promoter-TSS	Snurf	93.8	62.3
		chr19:30539701-30539800	36.4	promoter-TSS	Ppp1r2-ps3	92.5	28.2
		chr5:114806701-114806800	10.1	TTS	1500011B03Rik	96.6	57.4
		chr19:5756701-5756800	-10.87596	intron	Scyl1	64.4	34.9
		chr11:96712501-96712600	-11.663381	intron	Snx11	95.6	65.6
		chr17:26120801-26120900	-14.717965	intron	Mrpl28	96.4	65.8
		chr5:99613501-99613600	-10.970688	intron	A930011G23Rik	95.5	55.1
		chr1:134997401-134997500	-14.558143	intron	Ube2t	95.6	53.4
		chr6:128043701-128043800	-12.529061	intron	Tspan9	93.7	43.8
		chr2:20235701-20235800	-12.803134	Intergenic	Etl4	30.0	33.7
		chr9:119338101-119338200	-10.034581	exon	Myd88	66.7	34.5
		chr4:154341701-154341800	-12.870083	exon	Arhgef16	94.2	56.3
		chr1:34807101-34807200	-11.48459	exon	Arhgef4	97.3	45.2
		chr8:84701801-84701900	-10.958275	5' UTR	Lyl1	87.3	47.1

Supplementary Table S12: DAVID bioinformatics analysis showing the all enriched biological pathways for all genic DMTs in placenta and cortex localized to regions in previously published sequencing data with low methylation in sperm (≤10%) but moderate to high methylation in germinal vesicle oocytes (≥25%)and inner cell mass (≥25%).

Tissue	Exposure	Term	Count	PValue	Benjamini
Cortex	7FD	Signal Transduction	3	0.060	9.91E-1
		GTPase mediated signal transduction	2	0.076	9.50E-1
	10FS	Neuronal Action Potential	2	0.019	7.70E-1
	20FS	Dorsal/ventral Axis of Specification	2	0.013	9.05E-1
		Regulation of Wnt Signalling Pathway	2	0.018	8.04E-1
		Signal Transduction	5	0.032	8.63E-1
		Neuronal Action Potential	2	0.033	7.86E-1
Placenta	7FD	Multicellular Organism Development	6	0.026	9.98E-1
		Transcription, DNA-templated	7	0.086	1.00E0
		Negative Regulation of Wnt Signalling Pathway	2	0.089	1.00E0
	10FS	Glycine Biosynthetic Process	10	0.008	9.06E-1
		Transcription, DNA-templated	2	0.010	7.73E-1
		Positive Regulation of Transcription, DNA-templated	10	0.027	9.32E-1
		Regulation of Transcription, DNA-templated	5	0.031	9.05E-1
		Intracellular Steroid Hormone Receptor Signalling Pathway	7	0.033	8.66E-1
		Signal Transduction	2	0.036	8.36E-1
		Positive Regulation of Calcium Ion Transport	2	0.058	9.21E-1
	20FS	Protein Phosphorylation	5	0.009	9.18E-1
		positive regulation of NF-kappaB transcription factor activity	3	0.011	7.82E-1
		cytokine-mediated signaling pathway	3	0.019	8.31E-1
		response to lipopolysaccharide	3	0.032	9.04E-1
		cell proliferation	3	0.040	9.00E-1
